# Steroid Eluting Esophageal-Targeted Drug Delivery Devices for Treatment of Eosinophilic Esophagitis

**DOI:** 10.3390/polym13040557

**Published:** 2021-02-13

**Authors:** Alka Prasher, Roopali Shrivastava, Denali Dahl, Preetika Sharma-Huynh, Panita Maturavongsadit, Tiffany Pridgen, Allison Schorzman, William Zamboni, Jisun Ban, Anthony Blikslager, Evan S. Dellon, Soumya Rahima Benhabbour

**Affiliations:** 1 Department of Biomedical Engineering, UNC Chapel Hill & North Carolina State University, Chapel Hill, NC 27599-3290, USA; alkap@email.unc.edu (A.P.); roopalis@email.unc.edu (R.S.); ddahl@email.unc.edu (D.D.); panita@med.unc.edu (P.M.); 2 Department of Chemistry, University of North Carolina at Chapel Hill, Chapel Hill, NC 27599-3290, USA; 3 Division of Pharmacoengineering and Molecular Pharmaceutics, UNC Eshelman School of Pharmacy, University of North Carolina at Chapel Hill, Chapel Hill, NC 27599-3290, USA; preetika.sharma@unc.edu; 4 Department of Clinical Sciences, College of Veterinary Medicine, North Carolina State University, Raleigh, NC 27606, USA; tladams3@ncsu.edu (T.P.); anthony_blikslager@ncsu.edu (A.B.); 5 Division of Pharmacotherapy and Experimental Therapeutics, UNC Eshelman School of Pharmacy, University of North Carolina, Chapel Hill, NC 27599-3290, USA; aschorz@email.unc.edu (A.S.); zamboni@email.unc.edu (W.Z.); jsharie@email.unc.edu (J.B.); 6 UNC Lineberger Comprehensive Cancer Center, Chapel Hill, NC 27599-3290, USA; 7 Carolina Institute for Nanomedicine, Chapel Hill, NC 27599-3290, USA; 8 UNC Advanced Translational Pharmacology and Analytical Chemistry Lab, Chapel Hill, NC 27599-3290, USA; 9 Division of Gastroenterology and Hepatology, UNC School of Medicine, University of North Carolina, Chapel Hill, NC 27599-3290, USA; evan_dellon@med.unc.edu

**Keywords:** eosinophilic esophagitis (EoE), esophageal drug delivery systems, drug-eluting string, 3D printing, photopolymerizable resins, Drug loading strategies, steroids, fluticasone

## Abstract

Eosinophilic esophagitis (EoE) is a chronic atopic disease that has become increasingly prevalent over the past 20 years. A first-line pharmacologic option is topical/swallowed corticosteroids, but these are adapted from asthma preparations such as fluticasone from an inhaler and yield suboptimal response rates. There are no FDA-approved medications for the treatment of EoE, and esophageal-specific drug formulations are lacking. We report the development of two novel esophageal-specific drug delivery platforms. The first is a fluticasone-eluting string that could be swallowed similar to the string test “entero-test” and used for overnight treatment, allowing for a rapid release along the entire length of esophagus. In vitro drug release studies showed a target release of 1 mg/day of fluticasone. In vivo pharmacokinetic studies were carried out after deploying the string in a porcine model, and our results showed a high local level of fluticasone in esophageal tissue persisting over 1 and 3 days, and a minimal systemic absorption in plasma. The second device is a fluticasone-eluting 3D printed ring for local and sustained release of fluticasone in the esophagus. We designed and fabricated biocompatible fluticasone-loaded rings using a top-down, Digital Light Processing (DLP) Gizmo 3D printer. We explored various strategies of drug loading into 3D printed rings, involving incorporation of drug during the print process (pre-loading) or after printing (post-loading). In vitro drug release studies of fluticasone-loaded rings (pre and post-loaded) showed that fluticasone elutes at a constant rate over a period of one month. Ex vivo pharmacokinetic studies in the porcine model also showed high tissue levels of fluticasone and both rings and strings were successfully deployed into the porcine esophagus in vivo. Given these preliminary proof-of-concept data, these devices now merit study in animal models of disease and ultimately subsequent translation to testing in humans.

## 1. Introduction

Eosinophilic esophagitis (EoE) is an emerging chronic allergic disease characterized by eosinophilic infiltration of the esophageal mucosa [1,2]. The most common symptoms of EoE include dysphagia and food impaction in adolescents and adults, and abdominal pain, vomiting, heartburn, feeding intolerance, and failure to thrive in children. EoE represents a major healthcare burden as the estimated prevalence is at least 1 in 2000 Americans, and EoE-associated healthcare costs approach up to $1.4 billion per year [3,4]. Because there are no FDA-approved medications for EoE, asthma preparations, such as a fluticasone (FTS) from an inhaler, have traditionally been swallowed rather than inhaled to coat the esophagus [5]. The European Medical Agency (EMA) has approved a dissolvable budesonide tablet (Jorveza) for both acute and a long-term treatment. While these treatment approaches can be effective [6,7,8], because the medication delivery does not target the esophagus, it leads to suboptimal outcomes and adherence [9]. Our prior data, however, suggest that increased esophageal dwell time of pharmacologic agents is closely associated with subsequent treatment response [10]. To address these limitations, we aimed to produce strategies to more rationally provide drug exposure to the esophagus. Herein, we report the development of two esophageal-specific drug delivery platforms, a drug-eluting string and a 3D-printed ring for local rapid or sustained release of FTS in the esophagus.

These two delivery devices shared a common rationale—to deliver medication to the esophagus. A drug-eluting string could be swallowed, allowing a sustained release of drug along the entire length of the esophagus, perhaps during an overnight dwell, rather than the usual one-time bolus from a swallowed medication. While the minimally invasive esophageal string test has been used to absorb inflammatory factors related to EoE and characterize disease activity [11,12,13], there are no reports of delivering medication to the esophagus in this manner. A recent report outlined how a membrane deployed from a capsule might also target drug delivery to the esophagus. [14]. Our research strategy involved developing a FTS-eluting string and showing a target release of 1 mg/day of fluticasone in vitro for 24 h period of time. Fluticasone was coated on the surface of the string via drug adsorption using a dip coating process [15,16,17]. Studies were conducted to determine drug loading, in vitro drug release kinetics, ex vivo PK, and in vivo safety and PK in a porcine model [18].

In our second approach, we developed rings that can be inserted in the proximal esophagus to provide a long-acting sustained delivery of fluticasone. Rings were fabricated using 3D printing with additive manufacturing (AM), which involves a process of transforming digital models into real objects by placing materials layer by layer [19,20,21,22,23]. Recently, this emerging technology has been widely applied in the fields of drug delivery [24], biomaterials [20,25,26], medical [27] and food industry [28], and customized consumer products [29]. There are a number of 3D printing methodologies available which differ in how the 2D layers of materials are deposited. These include stereolithography (SLA) [30], two-photon polymerization [31], selective laser sintering (SLS) [32], fused deposition modeling (FDM) [33], and laminated object manufacturing (LOM) [34]. Among the existing AM technologies, photopolymerization offers the advantage of using a variety of synthetic polymers that can be tailored to have a desired structural property [35,36,37]. A photoprintable system mainly consists of an initiating system, photopolymerizable monomer or oligomer, and a light absorber [38]. Photoinitiators provide the required energy to initiate the photopolymerization, and a suitable light absorber is usually added to have an effective control over cure-depth [39]. The final properties of the printed part can be tailored by changing the constituents of photocurable resin formulation [40]. Recent advances in photopolymerization-based 3D printing involves spatially controlled curing of a photopolymer by interacting with a digitally modulated UV or visible light beam, defined by layer profiles of an Standard Tessellation Language (STL) file [41]. The process involves projection of an array of light spots on the surface of the resin, and propagation of light within each illuminated voxel in direction of the beam. The thickness of the photopolymerized layer is dependent on the interaction of photopolymer and the incident beam. In our system, we used digital light processing (DLP) projection as the method of illumination which has the advantages of high printing precision and fast printing speed [42]. 3D printing, which includes the ability to construct structures with complex geometries via computer-aided design (CAD) models, has opened the door for rapid and facile production of controlled drug release devices and polymer biomaterials [43,44,45,46,47].

In recent years, drug delivery devices have been developed to release medication at a controlled rate, and upon implantation, allow treatment without requiring oral or intravenous dosing [48,49,50]. We demonstrated in vitro that our novel 3D printed ring eluted fluticasone at a constant rate. All rings were fabricated using a variety of biocompatible photopolymerizable resin formulations, with various strategies of incorporating fluticasone in the ring device. Fluticasone-loaded rings were tested ex vivo and in vivo in the porcine model and showed high levels of fluticasone in the esophageal tissue in the ex vivo studies. To the best of our knowledge, these two approaches—the fluticasone-eluting string and ring—are among the first such esophageal-specific devices developed for the treatment of EoE. These promising results will need to be extended into disease models, with the ultimate goal of translating this technology into human disease.

## 2. Materials and Methods

### 2.1. Materials

Anhydrous dichloromethane (DCM), triethylamine (TEA), poly (ethylene glycol) Mn = 530 g mol^−1^, sodium chloride (NaCl), hydroxyethyl methacrylate (HEMA), diphenyl (2,4,6-trimethylbenzoyl)phosphine oxide (TPO), 2-tert-butyl-6(5-chloro-2H-benzo-triazol-2-yl)-4-methylphenol (BLS-1326), methacryloyl chloride, Solutol-HS 15, phosphate-buffered saline (0.01 M PBS, pH 7.4), HPLC-grade acetonitrile, water, and chloroform were purchased from Sigma Aldrich (St. Louis, MO, USA). Poly(ε-caprolactone) (PCL) (3 mm OD) string was purchased from Lactel absorbable polymers (Birmingham, Al, USA), and was made from poly (ε-caprolactone) ester terminated (Mn = 68 kDa). Cotton strings (1 mm OD) were kindly provided by Dr. Dellon. The therapeutic drug fluticasone propionate (FTS) was purchased from Letco medical (Decatur, Al, USA). All materials were stored as directed by the supplier and used as received.

HeLa (ATCC^®^ CCL-2™) cells were kindly provided by Dr. Chris Luft, University of North Carolina, Chapel Hill. HeLa cells were cultured in Dulbecco’s Modified Eagle’s Medium from Gibco Gaithersberg MD, USA) with 10% fetal calf serum from Gibco (Gaithersberg, USA) and 100 units/mL each of penicillin and streptomycin from Life Technologies (Grand Island, NY, USA). Cell lines were maintained at 37 °C in a humidified atmosphere containing 5% CO_2_, and ATCC guidelines were used for sub-culturing cells. For quantification of fluticasone in pig serum and esophageal homogenate in in vivo and ex vivo studies, pig plasma (K_2_EDTA anti-coagulant was purchased from BioIVT (Hicksville, NY, USA), fluticasone propionate, dimethylsulfoxide, and zinc sulfate solution were purchased from Sigma (St. Louis, MO, USA); d5-fluticasone propionate was purchased from Toronto Research Chemicals (North York, ON, CA); ammonium hydroxide, methanol, and acetonitrile were purchased from VWR (Radnor, PA, USA); and HLB μElution plates were purchased from Waters (Milford, MA, USA)

### 2.2. Fluticasone-Loaded Strings

#### 2.2.1. Optimization of Drug Loading

Poly(ε-caprolactone) (PCL) and fabric strings were cut into a length of 4 cm and marked up to a height of 2 cm (Supplementary Figure S1A). The dipping solution (2 mg/mL fluticasone in acetone/PLGA 1:5 *w/w*; 10 mL) was introduced in a 20 mL scintillation vial and the strings were immersed in the solution and incubated at room temperature for different time durations ranging from 1 min to 24 h (Supplementary Figure S1D). The strings were subsequently removed from the solution and allowed to dry at room temperature (RT) for 24 h. To investigate the effect of successive dipping of strings in the drug solution on percent drug loading, strings were subjected to a repeated dipping process. In this process, strings were incubated in the drug solution for a predetermined duration, removed, then reintroduced again in the solution and finally dried at RT. Various incubation times (1 min, 24 h), time between the repeated incubations (3, 10, 30 s), and number of incubation iterations (1 vs. 6) were investigated to optimize the amount of fluticasone loading onto the string.

#### 2.2.2. In Vitro Drug Release

To test the in vitro drug release, FTS-coated strings were individually placed in straight sided glass jars containing 20 mL of phosphate-buffered saline (PBS) (0.01 M with 2% Solutol HS, pH 7.4) under sink conditions at 37 °C. Sink conditions were defined as fluticasone (FTS) concentration at or below 1/5 of its maximum solubility (i.e., ≤0.11 mg/mL FTS) in PBS + 2% Solutol at 37 °C. The saturation solubility of FTS in PBS + 2% Solutol was determined by HPLC analysis. Sample aliquots (1 mL) were collected at 24 h time point, and FTS concentration was quantified by HPLC analysis. The amount of FTS released was quantified by HPLC analysis and was normalized to the total concentration of FTS coated on the string. All experiments were performed in triplicates.

#### 2.2.3. Ex Vivo Pharmacokinetic (PK) Studies

Drug elution from the strings was investigated ex vivo in a porcine model. In these studies, fresh normal esophageal tissue explants of pigs were used (Supplementary Figure S2A). These studies were performed on FTS coated strings with dimensions of 20 mm height and 2 mm cross-sectional diameter. Strings were inserted into approximately 20 mm of esophageal tissue section and incubated in 10 mL of PBS (to simulate the in vitro environment under sink conditions) at 4 °C for various durations (1 day and 3 days). A temperature of 4 °C was chosen for the study in order to preserve the fresh esophageal tissue. At each respective timepoint, the strings were removed, and the entire esophageal tissue was collected for PK analysis by LC-MS/MS analysis. In addition, residual FTS in the strings was extracted using acetonitrile and quantified by HPLC analysis.

### 2.3. Fluticasone Loaded Rings

#### 2.3.1. Synthesis of Poly(caprolactone dimethacrylate)

Poly(caprolactone-dimethacrylate) (PCL_700_-DMA) Mn = 700 gmol^−1^ was synthesized as described in the literature (Supplementary Scheme S1) [51]. In brief, poly-ε-caprolactone (PCL) diol (50 g, 96.22 mmol) was added to an oven-dried round-bottom flask. The reaction flask was sealed with a rubber septum and backfilled with nitrogen (N_2_). Anhydrous dichloromethane (DCM, 200 mL) and triethylamine (TEA, 222.5 mmol) were added and the flask was placed in an ice bath. A 10% molar excess of methacryloyl chloride (210 mmol) in 100 mL DCM was added dropwise at 0 °C over the course of 1 h and the reaction was stirred for 24 h at room temperature (RT). The TEA.HCl salt formed was removed by filtration and the filtrate was diluted 3 times with DCM, washed three times with a NaCl solution and three times with deionized water. The organic layer was collected, dried over magnesium sulfate, and filtered through a fritted funnel. DCM was removed under reduced pressure using a rotatory evaporator.

#### 2.3.2. Resin formulations for 3D Printing

For all experiments, a base resin containing no drug was formulated at the given ratios. Resin solutions with known amounts of photoinitiator, UV absorber, and reactive diluents dissolved directly into the liquid oligomeric monomers at different ratios. All resins were formulated by combining the components in an amber glass bottle and mixing with magnetic stirring overnight at room temperature.

#### 2.3.3. Device Design and Fabrication

3D-printed rings were fabricated using a top down, Digital Light Processing (DLP) Gizmo^®^ 3D printer (Gizimate^®^ 130, Queensland, Australia) equipped with a UV light source. Computer-aided design (CAD) files were downloaded from Thingiverse and modified using Meshmixer to design a ring suitable for the esophagus. The final dimensions of the ring CAD files were 24 mm outer diameter (OD), 20 mm height (H), and 2.5 mm cross section (CS). Standard tessellation language (STL) files were sliced at 50 μm thickness using the Gizmetor software, and printed at a continuous speed of 4 mm min^−1^ and a light intensity of 23 mW cm^−1^.

#### 2.3.4. Saturation Solubility of FTS in Resin Formulations

The saturation solubility of FTS was determined in all resin formulations used to print rings. For each resin formulation, 100 mg of drug was weighed into individual vials, and 200 mg of resin was added and allowed to stir at RT overnight to dissolve the drug. The mixture was then centrifuged for 15 min at 14,000 rpm (Eppendorf Centrifuge 5417C, Marshall Scientific, Hampton, NH, USA) to remove undissolved drug. Sample aliquots (1–2 mg, *n* = 4) were collected from the saturated supernatant and diluted with acetonitrile (ACN). Drug concentration in the saturated aliquots was determined by HPLC analysis.

#### 2.3.5. High-Performance Liquid Chromatography (HPLC)

A reversed-phase HPLC method was developed and validated to quantify the concentration of FTS released from strings and 3D printed rings. The HPLC analyses were carried out with a Thermo Finnigan Surveyor HPLA, (Thermo Finnigan, San Jośe, CA, USA) on an Intersil, ODS-3 column (4 μm, 4.6 Å ~150 nm (GL Sciences, Torrance, CA), maintained at 40 °C, with a flow rate of 1.0 mL/min, a 25 μL sample injection, and an acetonitrile/water mobile phase, each modified with 0.1% trifluoroacetic acid. A gradient method was utilized to achieve separation (0–20 min: 5–100% acetonitrile; 20–22 min: 100% acetonitrile; 23–25 min: 5% acetonitrile). Fluticasone was eluted at a retention time of 17.3 min. Data were collected at 265 nm and computed using Chromequest software. Concentrations were derived from a calibration curve generated using fluticasone standards prepared in 100% acetonitrile (250 μg/mL to 61 ng/mL).

#### 2.3.6. Gel Fraction and Percent Swelling

Swelling and gel fraction of 3D-printed rings was carried out in chloroform to determine the monomer incorporation and crosslink density. To determine gel fraction, rings (n = 3) were first weighed to record their initial mass at time zero (Mo) and then placed in 50 mL jars containing chloroform for one week. After one week, rings were removed and carefully cleaned with Kimwipe and the swollen mass (Ms) was recorded. The rings were subsequently air dried for three days and the dry weight (Md) was recorded. The gel fraction and degree of swelling was calculated using the following equations:

(1) $$\mathrm{Gel}\;\mathrm{Fraction}\;=\;\frac{\mathrm{Md}}{\mathrm{Mo}}$$

(2) $$\;\%\;\mathrm{Solvent}\;\mathrm{uptake}\;=\;\frac{\mathrm{Ms}\;-\;\mathrm{Md}}{\mathrm{Md}}\times \;100$$

#### 2.3.7. Rheology Analysis

The dynamic viscosity of various resin formulations (placebo and drug loaded) was measured using a Brookfield Cone and Plate Digital Rheometer (Model: LVDV-III + CP, Middleboro, MA, USA) and the reading was recorded at 25 °C with a spindle speed of 10 rpm.

#### 2.3.8. Mechanical Testing

The mechanical properties of 3D-printed rings were tested using Micro-strain analyzer TA instrument RSA III. The mechanical testing involved compression of the ring from the top in a radial fashion. The effect of post-fabrication UV cure on the mechanical properties of 3D printed rings was investigated. Rings were fabricated with biodegradable resins (PCL_700_-DMA, PCL_700_-DMA/HEMA) and tested in triplicates to determine the compressive force. The average force required to achieve 10%, 20%, and 50% compression of the ring diameter was measured at the proximal, center, and distal ends of the rings. Additionally, the rings were rotated clockwise at three different angles to get the average compression force value of the entire ring.

#### 2.3.9. Drug Loading Studies

**Figure 1:**
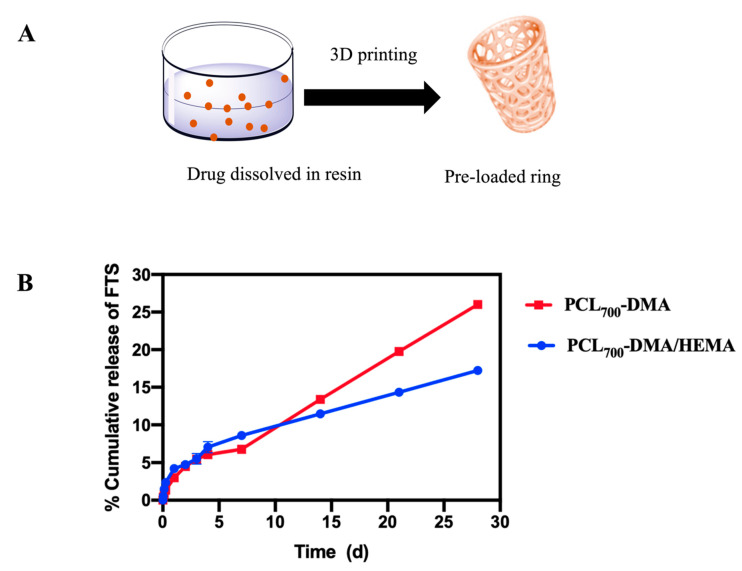
Effect of crosslink density of resin formulations on drug release kinetics (**A**) A pictorial representation of pre-loading FTS into 3D printed rings. (**B**) In vitro release kinetics of FTS-loaded rings incubated in PBS at 37 °C for 30 days. All error bars represent standard deviation for *n* = 3.

FTS-loaded rings were fabricated using a pre-loading or post-loading process. In the pre-loading method, the drug was added into the resin precursor at a given weight % (wt %) and stirred at room temperature overnight to fully dissolve the drug (Figure 1). In the post-loading process, drug was incorporated into the ring post 3D printing ring fabrication (Figure 3). Placebo rings were fabricated with a base resin formulation using the 3D printing process described above. Pre-weighed placebo rings were incubated in acetone (50 mL) at RT for 24 h to remove unreacted resin components (leachables/extractables) from the rings. The rings were subsequently air dried overnight, and their final weight recorded. The dried rings were then incubated in a saturated solution of FTS in acetone at RT for 24 h. The drug was absorbed into the polymer network via swelling of the ring matrix in the concentrated drug solution. The post-loaded rings were subsequently air-dried overnight at RT to remove all solvent and the final mass of rings was recorded.

#### 2.3.10. In Vitro Cumulative Drug Release

To test the in vitro drug release, FTS-loaded rings (pre- and post-loaded) were individually placed in straight sided glass jars containing 200 mL of PBS (0.01 M with 2% (*w/w*) Solutol, pH 7.4) and incubated at 37 °C under sink conditions for 30 days. Sample aliquots (1 mL) were collected at various time points and replaced with fresh release medium. The release medium was completely removed and replaced with fresh medium every week to maintain sink conditions. FTS concentration in release samples was quantified by HPLC analysis. Cumulative drug release was normalized to the total concentration of drug in the ring determined by HPLC analysis. All experiments were performed in triplicates.

#### 2.3.11. In Vitro Cell Viability Studies

The safety and tolerability of the resin materials used to fabricate the rings was tested in vitro in HeLa cells. The HeLa cell line is the most robust and widely used cell line to assess in vitro cell viability [52]. HeLa cells were plated at a density of 17,500 cells/well in 96-well opaque, flat-bottomed, white plates from Corning Inc. (Corning, NY, USA) 48 h prior to treatment. The cell lines were treated with 3D-printed small discs (Supplementary Figure S4A). Cell viability was assessed after a 48 h treatment exposure using CellTiter-Glo^®^ Luminescent Cell Viability Assay from Promega (Madison, WI, USA) and plates were read using the Synergy 2 Multi-mode Plate Reader from BioTek Instruments (Winooski, VT, USA). The CellTiter-Glo Luminescent Cell Viability Assay is a homogeneous method to determine the number of viable cells in culture based on quantitation of the ATP present (*Renilla* luciferase units (RLU)), which signals the presence of metabolically active cells. Cells grown in growth media alone were used as controls. Cell viability was calculated as a percentage of RLU Experimental/RLU Control.

#### 2.3.12. Ex Vivo Pharmacokinetic (PK) Studies

3D-printed rings were tested ex vivo in fresh porcine esophagus to determine the concentration of FTS released into the esophageal tissue overtime. These studies were performed with both pre- and post-loaded FTS-loaded rings (10 mm H, 10 mm OD, and 2 mm CS) (Supplementary Figure S5A). Drug-loaded rings were inserted into approximately 10 mm of esophageal tissue explants. The esophageal tissue explants were subsequently placed in 10 mL of PBS at 4 °C and incubated for 1, 3, 7, and 14 days (*n* = 3 per timepoint). At each timepoint, the rings (*n* = 3) were removed from the media and the esophageal tissue was collected and stored at −80 °C for pharmacokinetics analysis by LC-MS/MS. To determine the total amount of fluticasone delivered to the esophageal tissue ex vivo, fluticasone released in PBS and residual drug in the ring were quantified by HPLC analysis.

### 2.4. In Vivo PK Analysis by LC-MS/MS

#### 2.4.1. Sample Preparation—Solid-Phase Extraction of Serum and Plasma Samples

Blood was harvested and centrifuged to isolate the serum, which was stored at −80 °C until time of analysis. Fluticasone stock solutions (1 mg/mL) were prepared and stored in DMSO at −80 °C. The standard curve and quality controls (QCs) were prepared by spiking fluticasone intermediate solutions prepared in methanol into K2-EDTA pig plasma to final concentrations of 10, 30, 50, 100, 300, 500, 1000, and 3000 pg/mL and 25, 250, and 2500 pg/mL, respectively. It was determined that plasma served as a surrogate matrix for serum for measurement of fluticasone. The quantitative method described here was adapted from a previously published method [53]. In short, 300 μL of 40 mM ZnSO_4_/10% ammonium hydroxide was added to 300 μL of plasma standard, QC, blank, or serum sample. The mixture was vortexed briefly and centrifuged at 10,000× *g* for 15 min. An HLB μElution plate was conditioned with 100 μL methanol and equilibrated with 2 × 200 μL ddH_2_O before 550 μL of plasma or serum supernatant was loaded to the appropriate well of the plate. Each well was washed with 2 × 200 μL of 25% methanol and the fluticasone eluted into a polypropylene 96-well plate with 2 × 50 μL of acetonitrile: methanol (1:1). The eluent was diluted with 150 μL ddH_2_O before analysis by LC-MS/MS.

#### 2.4.2. Protein Precipitation of Esophagus Tissue Homogenate

Proximal, mid, and distal sections of esophagus tissue were frozen after harvest, as above, and stored at −80 °C prior to homogenization. Representative pieces of tissue were excised from each section, weighed, and homogenized in PBS (1:3) using three 30 s cycles at 5500 rpm with six 2.8 mm ceramic beads in a bead mill homogenizer (VWR). Homogenates were stored at −80 °C until time of analysis. The standard curve and QCs were prepared by spiking fluticasone propionate intermediate solutions prepared in methanol into esophagus homogenate from untreated animals at 1, 3, 5, 10, 30, 50, 100, 300, 500, 1000, 3000, and 5000 ng/mL and 4, 40, 400, and 4000 ng/mL, respectively. Each 50 μL standard, QC, blank, or sample was extracted with 200 μL of acetonitrile containing 5 ng/mL of d_5_-fluticasone propionate internal standard. Samples were vortexed for 5 min, centrifuged at 10,000× *g* for 10 min at 4 °C. The supernatant containing fluticasone propionate was transferred to a clean tube and evaporated under nitrogen at 40 °C. Samples were reconstituted in 500 μL of 25% methanol and analyzed by LC-MS/MS.

#### 2.4.3. LC-MS/MS Analysis

Liquid chromatography for fluticasone propionate was accomplished using a Shimadzu LC-20AD liquid chromatograph with an XBridge BEH C18 (2.5 μm 2.1 × 50 mm) analytical column (Waters) protected with a guard column. The mobile phase consisted of 0.1% ammonium hydroxide (mobile A) and methanol (mobile phase B), and used a gradient of 50% to 95% mobile phase B over 1.5 min. The flow rate was 0.3 mL/min and the total run time was 5 min. Compounds were detected using a Thermo TSQ Ultra triple quadrupole mass spectrometer equipped with a heated electrospray ionization source in the positive ion mode. The spray voltage was held at 4.0 kV and the vaporizer temperature at 300 °C. Fluticasone propionate and d_5_ fluticasone propionate (internal standard) were detected by multiple-reaction monitoring (MRM) using the transitions 501 ≥ 293 *m/z* and 506 ≥ 293 *m/z*, respectively. Calibration curves were fit using linear regression with 1/X^2^ weighting in Xcalibur^®^ v. 2.0 (Thermo Fisher Scientific, Waltham, MA, USA).

### 2.5. In Vivo Pharmacokinetic (PK) Studies

All in vivo animal studies were approved by the North Carolina (NC) State IACUC. Yorkshire-cross pigs (~25–35 kg bodyweight to allow an optimal esophagus size approximating a human esophagus size) were obtained from campus-maintained breeding herds at NC State University’s Swine Educational Unit, and then transferred to the care of Laboratory Animal Resources (LAR) at the College of Veterinary Medicine (CVM). CVM facilities are AAALAC- and USDA-approved, and are managed by the University Attending Veterinarian and a team of board-certified Laboratory Animal Veterinarians (LAVs), Registered Veterinary Technicians (RVTs), and animal care workers. Endoscopy was performed with the pigs under general anesthesia with assistance from RVTs for anesthesia, using standard of care protocols. Pigs were sedated with a combination of xylazine (0.25 mg/kg) and ketamine (11 mg/kg) administered IM, followed by mask induction of anesthesia with isoflurane vaporized in 100% O_2_. Pigs were then orotracheally intubated and maintained on isoflurane throughout the procedure. All post-endoscopic care of the animals was performed by LAVs, RVTs, and animal caretakers within LAR, in accordance with protocols established in the IACUC application. Any evidence of postoperative pain was managed with administration of buprenorphine (0.05 mg/kg, IM, q8h as needed).

For endoscopic ring implantation, three sets of 5 pigs were used. In the first set, 3D-printed rings were used without fluticasone (placebo rings) to confirm that the rings could be placed. In order to deploy the rings, the rings were back-loaded onto the inner section of an esophageal overtube (Gardus, Steris Healthcare, Mentor, OH, USA), through which the endoscope was inserted and the esophagus was intubated with the tube and ring in place. Then, the outer section of the overtube was gently advanced over the inner tube to push the ring into position in the proximal esophagus. As this technique was successful, fluticasone-eluting rings were deployed in the two additional sets of pigs. Rings were allowed to indwell for seven days during which time the pigs were provided with an ad lib diet, and then repeat endoscopy was performed to determine if the ring was still seated at day seven, serum, and plasma specimens were obtained for PK analysis and the animals were euthanized per the standard CVM/LAR protocol consistent with 2009 American Veterinary Medical Association (AVMA) Guidelines on Euthanasia. Pigs were initially sedated as before with xylazine/ ketamine, after which they were administered an overdose of pentobarbital (60 mg/kg, IV). The esophagus was immediately explanted once euthanasia had been confirmed, divided into proximal, mid, and distal segments, and flash-frozen for subsequent PK analysis.

For the endoscopic string implantation, an additional three sets of 5 pigs were used. For the first set, the thicker fluticasone-coated string was advanced through a previously placed esophageal overtube, and then held in place as the overtube was removed. The string was then brought through the posterior aspect of the pig’s cheek (caudal to the last molar) after making a small incision and retrieving the string with forceps. Strings were externally fixed in place with 2-0 nylon suture and covered with a section of iodine-impregnated self-adhering drape material. The string was allowed to dwell for 24 h while solid feed was withheld, and buprenorphine analgesia was provided. At 24 h, the animals were euthanized, and serum, plasma, and esophageal samples were obtained as above. The procedure for the second set of pigs was identical to the first, with the exception of a 72 h string dwell time, and after the first 24 h the pigs were allowed to eat a liquid diet. For the last set of pigs, the FTS-impregnated fabric string was endoscopically placed. For this, biopsy forceps were used to grasp a fabric tape tag at the end of the string, and gently advanced this into position using direct visualization. After external fixation as above, this string had a 24 h dwell time, with the same sample procurement protocol as for the first set of pigs.

## 3. Results

### 3.1. Fluticasone-Eluting Esophageal String

#### 3.1.1. Dip Coating Parameters Optimization

In the dipping process, the amount of drug coated onto a device can be optimized by balancing the viscosity of the drug solution, the incubation and drying times, and the number of dipping/drying iterations [54,55]. A comprehensive study was carried out to investigate the aforementioned effects on the amount of fluticasone loaded onto the string. Fluticasone (FTS)-loaded strings were designed to cover the entire human esophagus length (~25 cm) and release a clinically relevant dose of FTS (1–2 mg) in 24 h [54]. Initial optimization studies were accomplished with a commercially available biocompatible PCL string. The coating solution was comprised of FTS in a biocompatible polymer and organic solvent. The solvent used to load FTS onto the PCL string using a dip-coating process was selected based on high solubility of FTS in the solvent, its volatility, and ease of removal post drug loading. A number of solvents were screened, and acetone was selected as a dipping solvent based on its low boiling point and high saturation solubility of FTS (~20 mg/mL). To optimize the dip-coating process, poly(lactide-*co*-glycolide) (PLGA, 50:50) of various molecular weights (MW 11, 27, and 53 kDa) was used as a model to investigate the effect of dipping solution viscosity on percent PLGA loaded onto the string (Supplementary Figure S1D). Results from these experiments were used to extrapolate the amount of FTS that can be loaded on the strings using the drug-loaded dipping solution and minimize the use of an expensive drug for preliminary optimization studies.

A series of control experiments were performed to evaluate the effect of polymer molecular weight (MW) on the mass of polymer loaded onto strings under the same dipping conditions. The prototype PCL strings (3 mm OD, 20 mm L, *n* = 3), were pre-weighed (M_i_) before the dip coating process. The dipping conditions involved incubating PCL strings in the dipping solution for 1 min followed by drying at RT for 24 h. After drying, the strings were weighed again (M_f_) and mass of polymer loaded was calculated as the difference in mass (M_f_ − M_i_) (Supplementary Figure S1D). Results showed that the amounts of PLGA loaded onto the string increased with increasing the dipping solution viscosity. The viscosity of the dipping solution significantly increased with increasing PLGA MW from 1.4 cP (PLGA MW 11 kDa) to 15.7 cP (PLGA MW 53 kDa). This increase in viscosity resulted in a 2.67-fold increase in PLGA loading onto the string (4.67 ± 0.09 vs. 1.75 ± 0.17 mg PLGA) with the highest viscosity solution (15.7 cP) compared to the lowest viscosity solution (1.4 cP). To investigate the effect of incubation time on PLGA loading, strings were incubated in PLGA solutions at RT for 1 min or 24 h (Supplementary Figure S1D). Results showed that there was no significant difference in PLGA loading with longer incubation time. The number of dipping iterations on PLGA loading was investigated over a 1 min incubation period. Strings were dipped once and incubated for the entire 1 min in PLGA solutions or dipped for 6 consecutive times (10 s each) over 1 min. Results showed that increasing the number of dipping iterations resulted in a significant increase in PLGA loading onto the strings for all three PLGA MWs. PLGA loading increased from 1.75 mg to 2.61 mg, 3.07 to 4.6 mg, and 4.67 to 7.27 mg for PLGA MW 11, 27, and 53 kDa, respectively (Supplementary Figure S1D). Finally, the effect of drying time between iterative dipping on PLGA loading was investigated. Results showed that increasing the drying time between iterative dips from 3 s to 30 s resulted in a significant increase in PLGA (MW 53 kDa) loading from 4.67 mg to 10.73 mg (Supplementary Figure S1E). Based on the collective data from the aforementioned optimization steps, the optimized drug loading conditions using the dip-coating process were set to a total of 1 min incubation time with 6 consecutive dipping steps (10 s each) and 30 s drying time between consecutive dipping steps. The loading solution used in subsequent studies with fluticasone (FTS) consisted of 1:5 *w/w* PLGA/acetone. The saturation solubility of FTS in the dipping solutions was ~10 mg/mL for solutions made with PLGA MW 11 and 27 kDa and ~6 mg/mL in the solution containing the highest MW PLGA (53 kDa) (Supplementary Figure S1E).

As the string would ultimately be for human use with an average esophageal length of 20–25 cm (the same length as in the porcine model), and for an overnight dwell time, the drug release was tested for a period of 24 h. Optimization studies were carried out with a 2 cm PCL string and extrapolated to a 25 cm string based on the homogenous and consistent drug loading data obtained from the optimization studies (Table 1). In vitro release studies showed that FTS had a slow burst release within 24 h (≤1%) corresponding to ~15, ~23, and ~44 µg for solutions containing PLGA MW 10, 27, and 53 kDa, respectively. These FTS concentrations were much lower than a targeted release of 1–2 mg FTS in 24 h (Table 1). The low FTS burst release within 24 h was attributed to a high affinity of FTS to PLGA given its hydrophobic nature (LogP = 2.78) resulting in a slow diffusion from the polymer layer to the release medium. To enhance FTS release from the strings, PLGA was removed from the loading solution and a saturated solution of FTS in acetone (20 mg/mL) was used. The drug loading process using the saturated FTS solution in acetone was further optimized to include a 4 min incubation time, with 24 consecutive dipping steps and 30 s drying steps between each consecutive dipping steps. These new loading parameters resulted in significantly higher FTS release within 24 h meeting the targeted 1 mg/day release. This optimized process was used to test FTS release both ex vivo and in vivo in a porcine model.

To design a clinically translatable fluticasone eluting string for human use and easy swallowing, a fabric (cotton) string was loaded with FTS using the optimized dip-coating process (4 min, 24 dips, and 30 s drying) and investigated for FTS release in vitro, ex vivo, and in vivo. In vitro release studies showed that FTS was released from the fabric string at the target rate with ~1.4 mg/day for a 25 cm string (Table 1).

#### 3.1.2. In Vivo Pharmacokinetic (PK) Studies

In vivo studies were carried out in a porcine model to assess the local and systemic pharmacokinetics of FTS from drug-eluting strings (Supplementary Figure S6). FTS-loaded strings (25 cm L) were prepared using the dip-coating method described above. The strings were placed along the entire length of the pig’s esophagus as described above. FTS level was measured in both esophagus tissue and plasma using LC-MS/MS analysis. The serum samples collected at 1 and 3 days post string administration showed minimum FTS concentrations (FTS < 30 pg/mL). There was only one exception of FTS concentration (57.6 pg/mL) detected in serum sample of one pig at day 3 post string administration. In contrast, FTS was detected in all in vivo esophageal tissue explants with higher FTS concentrations detected in day 1 (105.89 ±10.87 µg) compared to day 3 (14.42 ± 9.9 µg) (Supplementary Figure S6B) for FTS-loaded PCL strings. Ex vivo studies with fluticasone coated PCL strings (2 cm L) on a fresh porcine esophageal section incubated for 1 or 3 days in PBS showed an opposite trend to in vivo studies with an increase in FTS tissue accumulation after 3 days incubation in PBS (102.2 ± 27.6 µg) compared to a 24 h incubation (31.78 ± 13.19 µg) (Supplementary Figure S2B). These results demonstrate that FTS was released from the strings at a continuous rate and was highly accumulated in the esophageal tissue both ex vivo and in vivo with minimal or no accumulation in the blood in vivo.

To evaluate a more clinically relevant string material, additional in vivo PK studies were carried out with FTS-loaded fabric strings in the porcine model (Supplementary Figure S6A). An average of 22.08 ± 0.31 (*n* = 5) mg fluticasone was loaded on 25 cm long (L) strings. Strings were then implanted in the esophagus as described above for 1 day of dwell time. Blood (plasma) samples collected at 1 day showed no absorption of FTS, with an exception of 106 pg/mL detected in one pig. FTS was detected in 4 out of 5 pigs’ blood (serum) samples with an average of 83 ± 21.6 pg/mL (*n* = 4). On the other hand, FTS was detected in all in vivo esophageal tissue explants at significantly higher concentrations of 23 ± 12.9 µg, which is orders of magnitude higher compared to serum levels. These results showed that FTS accumulated in the porcine esophageal tissue explants at high levels (~2 × 10^5^ serum levels) detected at each collection time point, with minimum systemic absorption of FTS detected in blood (serum/plasma) samples. To corroborate the in vitro release studies and in vivo PK studies, ex vivo studies were performed with FTS-loaded fabric strings (2 cm L, *n* = 3) to determine FTS accumulation in porcine esophagus tissue explants over 24 h incubation in PBS at 4 °C (Supplementary Figure S2). An average of 103.9 ± 35.4 µg FTS was accumulated in the esophageal tissue explants, which corroborates the in vivo results demonstrating the absorption of FTS in the esophagus.

### 3.2. Fluticasone-Eluting Esophageal 3D Printed Rings

Fluticasone-loaded esophageal rings were fabricated using biocompatible resins. PCL is commonly used for biomedical applications owing to its biocompatibility and robust mechanical properties [56,57,58,59]. To fabricate esophageal rings PCL was chemically functionalized to introduce methacrylate groups and enable photopolymerization cross-linking during the 3D printing process via radical-mediated cross-linking reactions. PCL-diol was successfully functionalized with methacrylate groups to produce PCL dimethacrylate (PCL-DMA) using a previously reported method (Supplementary Scheme S1) [51]. The structure of PCL-DMA was confirmed with nuclear magnetic resonance (NMR) analysis by the presence of peaks at δ5.8 ppm (1H) and δ6.1 ppm (1H), corresponding to the vinyl protons and a peak δ1.5 ppm corresponding to methyl groups (3H) of the methacrylate moiety (Supplementary Figure S3).

Fluticasone (FTS) was formulated in the rings via a pre-loading or post-loading process. In the pre-loading process, FTS was dissolved in a liquid resin prior to 3D printing fabrication. The resin formulation composed of a photoinitiator (TPO), UV absorber (BLS), and PCL_700_-DMA added at predetermined ratios (Table 2) [51]. The mole percent (%) of the various resin components was calculated relative to the methacrylate groups. The resin components were mixed and stirred overnight in an amber glass bottle at room temperature (RT). Resin viscosity in the range of 200 to 600 cP was required in order to allow fabrication accuracy and part shape fidelity using the 3D printing process [60,61]. For photopolymerization-based 3D printing, a light reactive diluent is usually added to reduce the viscosity of the resin [59,62]. Resin formulations used to fabricate the rings had viscosities in the aforementioned printable range. The viscosities of resin formulations were 490 cP and 350 cP for PCL_700_-DMA resin without diluent and PCL_700_-DMA with a diluent (HEMA) respectively.

The saturation solubility of FTS in the PCL_700_-DMA and PCL_700_-DMA/HEMA resin formulation was determined by HPLC analysis and quantified at 14.62 ± 0.82 mg/g and 12.91 ± 0.09 mg/g, respectively. To ensure FTS was homogenously dissolved within the resin, sample aliquots (*n* = 4) were collected from different areas in the solution and analyzed by HPLC. The FTS-resin formulation was deemed homogenous if the average concentration of FTS in the sample aliquots (*n* = 4) had a standard deviation ≤5%. The final resin formulations contained 1.4% *w/w* FTS in the resin. FTS-loaded rings were fabricated using 3D printing as described above and the amount of FTS loaded in 3D-printed rings was determined by incubating rings in acetonitrile (ACN, 50 mL) at 37 °C for 24 h to extract FTS and quantifying FTS concentration in ACN by HPLC analysis.

The stability of FTS in the resin formulations (PCL_700_-DMA and PCL_700_-DMA/HEMA) was determined under three storage conditions of varying temperature and relative humidity (RH) (25 °C, 25 °C/60% RH, 40 °C/75%RH) over 6 months by quantifying FTS concentration in the resin by HPLC analysis. Results showed that FTS was stable under all three storage conditions for up to 6 months (Supplementary Figure S7).

#### 3.2.1. In Vitro Release Studies

In order to achieve the target drug release rate of 1 mg/day over 30 days, rings were loaded with 30 mg of FTS (Figure 1A). The average amount of drug loaded in the rings was 32.11 ± 1.21 mg as determined by drug extraction and HPLC analysis. Results showed FTS had a minimum burst release of ~3% (958 μg) in the first 24 h followed by zero-order kinetics at 282 μg/day over 30 days (Figure 1B, Table 3). The daily drug release from these rings was lower than the target drug release (1 mg/day). An important advantage of utilizing 3D printing is the ability to use a variety of photopolymerizable resins and ring designs. The rate of diffusion and drug release can be controlled by changing the crosslink density of the resin as well as the chemical structure and properties of the polymer. Hydroxyethylmethacrylate (HEMA) was added as a hydrophilic chain extending diluent to study the effect of crosslink density of resin formulation on the release kinetics of FTS (Table 2).

Results showed that FTS exhibited a minimum burst release in the first 24 h (~4.5%, 1.31 mg) and slower zero-order kinetics with 207.0 μg/day over 30 days compared to 282 μg/day obtained with FTS rings fabricated without HEMA (Table 3). The slower release kinetics when HEMA was included as a diluent in the resin was attributed to the presence of hydrogen bonding between the hydroxyl groups of HEMA and the carboxyl groups in FTS (Supplementary Figure S8). The hydrogen bonding resulted in a tighter association between the drug and the polymeric network of the resin formulation and thus slower release rate in vitro. Collectively, these results showed that FTS release kinetics could be fine-tuned by changing the resin composition.

#### 3.2.2. Mechanical Properties of 3D Printed Rings

To enable successful implantation in the esophagus, the mechanical properties of the FTS-eluting esophageal rings were tested and optimized. In general, a post-fabrication UV or thermal curing process is implemented in 3D printing in order to improve the mechanical properties of 3D printed parts [63,64,65,66,67,68]. These post-fabrication curing treatments lead to improved compression strength and elastic modulus of the 3D-printed parts [69,70,71]. The effect of post-fabrication UV curing on the mechanical properties of placebo rings fabricated without (PCL_700_-DMA) or with diluent (PCL_700_-DMA/HEMA) was investigated. UV-treated and non-UV-treated rings were compressed to a distance of 10, 20, and 50% of their outer diameter. The load at these percent compressions in Newtons (N) was reported (Table 4). All measurements were done in triplicates. Results showed that using both resins (PCL_700_-DMA, PCL_700_-DMA/HEMA), rings exposed to UV treatment post fabrication had significantly higher compression forces at all compression distances compared to rings that did not undergo post-fabrication UV treatment (Table 4).

The Young’s modulus measures the resistance of a ring to elastic deformation under a specific load and its ability to recover its original shape once the stress force is removed. Results showed that rings exposed to UV curing post fabrication were stiffer and had a higher Young’s modulus (1736.33 ± 34.8 Pa for PCL_700_-DMA and 1830.9 ± 60.3 Pa for PCL_700_-DMA/HEMA) compared to 8.61 ± 3.5 Pa (PCL_700_-DMA) and 10.93 ± 1.2 Pa (PCL_700_-DMA/HEMA) for rings that did not undergo post-fabrication UV cure (Supplementary Table S1). Collectively, these results demonstrate that UV curing post fabrication significantly improved the mechanical properties of 3D printed rings. The increase in compression force and Young’s modulus was attributed to an increase in crosslink density of the polymer matrix upon exposure to UV and formation of a tighter network. This effect was confirmed by determining the gel fraction and percent (%) swelling of the rings in organic and aqueous solvents. Rings that were exposed to a UV curing treatment post fabrication had higher gel fraction and lower percent swelling compared to rings that did not undergo UV curing (Supplementary Table S1) due to higher crosslink density of the polymer matrix as a result of UV exposure.

**Figure 2:**
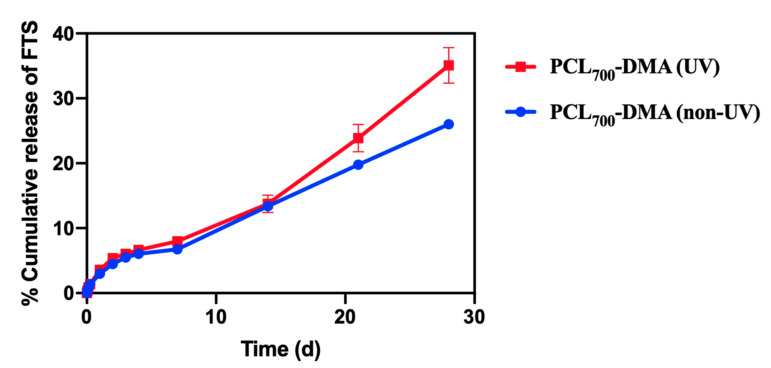
In vitro release kinetics of FTS from non-UV- and UV-cured rings printed with PCL_700_-DMA resin formulation. Rings were incubated in PBS at 37 °C for 30 days and sample aliquots (1 mL) were collected and analyzed by HPLC analysis. All error bars represent standard deviation for *n* = 3.

To assess the stability of FTS when exposed to post-fabrication UV curing, in vitro release kinetics of fluticasone from UV cured rings was investigated (Figure 2). Results showed that rings exposed to a post-fabrication UV cure had slightly higher burst of FTS in the first 24 h (4%, 1179.9 μg) and higher zero-order release rate (370 μg/day) compared to non-UV cured rings (Table 5). These results showed that UV curing improved the mechanical properties of rings and resulted in higher release rates of FTS in vitro.

#### 3.2.3. Effect of Drug Incorporation Process

**Figure 3:**
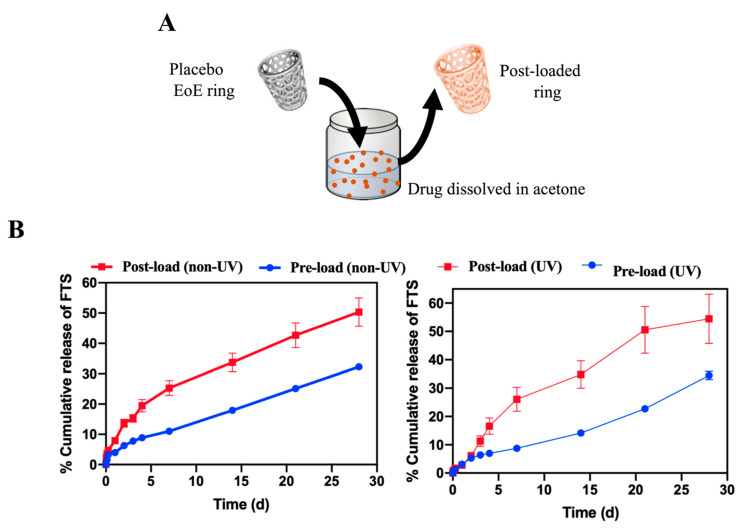
Effect of drug incorporation process onto drug release kinetics of rings printed with the PCL_700_-DMA resin formulation. (**A**) A pictorial representation of post-loading of FTS into 3D printed placebo ring. (**B**) In vitro release kinetics of FTS from pre-loaded (non-UV- and UV-cured rings) and post-loaded (non-UV- and UV-cured rings) printed using the PCL_700_-DMA resin formulation. Rings were incubated in PBS at 37 °C for 30 days and sample aliquots (1 mL) were collected and analyzed by HPLC analysis. All error bars represent standard deviation for *n* = 3.

Fabrication of drug-loaded rings can be done by either incorporating drug(s) in the initial resin formulation (termed pre-loading, Figure 1A) or by adding drug(s) to a 3D printed ring post fabrication (termed post-loading, Figure 3A). We sought out to investigate the effect of FTS incorporation step on in vitro release kinetics from rings. A number of solvents were screened to determine a suitable solvent for post-loading FTS, and acetone was selected based on high solubility of FTS and ease of removal. Placebo rings were post-loaded with FTS by incubating them in a near saturated solution of FTS in acetone (20 mg/mL; 50 mL) at RT for 24 h.

Results demonstrated that rings post-loaded with FTS exhibited ~2-fold increase in burst release within the first 24 h (~8%, 550 μg) compared to rings pre-loaded with FTS (~4%, 280 μg). Rings post-loaded with FTS also exhibited greater zero-order release kinetics over 30 days, with 90 μg/day compared to 50 μg/day for pre-loaded rings (Figure 3B, Table 6). These results demonstrate the ability to fine-tune drug release kinetics using different drug loading processes.

Similar to the pre-loaded rings, the effect of post-fabrication UV curing on fluticasone release rate in post-loaded rings was investigated. 3D-printed placebo rings were UV cured using the method described above and subsequently incubated in a near saturated solution of fluticasone in acetone at RT for 24 h. Results showed that post-loaded UV-cured rings exhibited a lower burst release of FTS in the first 24 h (~3%, 420 μg) but faster zero order release rates ~270 μg/day over 30 days compared to non-UV cured post-loaded rings (Table 6). For both drug loading strategies, fluticasone exhibited a sustained zero-order release profile over 30 days, demonstrating the potential use of 3D printed rings as a long-acting esophageal drug delivery device.

#### 3.2.4. In Vitro Cytotoxicity Studies

Biocompatibility of 3D-printed rings was assessed in vitro in HeLa cells. Small discs (3 mm OD) were fabricated with PCL_700_-DMA resin and tested with HeLa cells in 96-well plates. Placebo, FTS-loaded, and UV-cured FTS-loaded discs were tested for cytotoxicity using the CellTiter-Glo^®^ luminescent viability assay. Results showed that all discs were biocompatible and well tolerated by the HeLa cells (Supplementary Figure S2).

#### 3.2.5. Ex Vivo Pharmacokinetic Studies

To assess the pharmacokinetics of FTS in local tissue, ex vivo studies were performed using fresh porcine esophageal tissue with pre-loaded and post-loaded fluticasone rings (*n* = 3 per timepoint). The FTS concentration in esophageal tissue was determined using LC-MS/MS. Results demonstrated that sustained levels of FTS were detected in all samples over 14 days (Supplementary Figure S3). These results demonstrate that FTS was effectively diffused from the rings and highly accumulated in the esophageal tissue.

#### 3.2.6. In Vivo Pharmacokinetic Studies

Additionally, in vivo studies were carried out in a porcine model to assess the local and systemic pharmacokinetics of FTS-loaded rings. To ensure that rings were properly placed in the proximal part of the esophagus, initial studies were carried out using placebo rings (without FTS). We have shown successful endoscopic ring implantation into the proximal esophagus of one set of five pigs. After successful implantation of placebo rings, FTS-loaded rings were fabricated using pre-loaded PCl_700_-DMA resin formulation. Additionally, a post-fabrication UV curing was implemented to fabricate rings with mechanical properties appropriate for esophageal implantation. These FTS-loaded rings were deployed in the proximal esophagus of two sets of five pigs each for a period of 7 days. A daily endoscopy was performed to monitor the placement of rings. During endoscopic screening, it was observed that the rings were not present in the proximal esophagus of pigs after day 1. This observation was corroborated by the absence of FTS in esophageal tissue and plasma collected at euthanasia at day 7 post ring administration. As the pigs were provided with an ad lib diet during the study, we hypothesize that the rings slipped into the pig’s stomach along with the food. The PK analysis showed no accumulation of FTS in the esophageal tissue and no detectable plasma levels at day 7. These studies demonstrated that rings can be successfully inserted in the proximal part of the esophagus; however, further optimization in order to keep these rings in place for a longer time is needed.

## 4. Discussion

Development of new and innovative esophageal medication delivery systems are urgently needed for the treatment of EoE. In this study, we provide a comprehensive overview of the development of two types of drug delivery systems: (1) an FTS-eluting string for a rapid but sustained overnight release, and (2) a 3D-printed FTS-eluting ring to allow a long-acting constant drug release. In vitro release studies of FTS from drug-coated strings achieved the target drug release for a 24 h time period. FTS-eluting 3D-printed rings are capable of providing drug release for a period of 1 month or longer in vitro. FTS can be incorporated into the rings via two drug loading processes. Pre-loading involves addition of FTS into the resin formulation prior to ring fabrication. FTS loaded in these resin formulations was stable under accelerated stability storage conditions over six months, and FTS retained its physical and chemical properties. Our results have suggested that this method of drug loading exhibited controlled and sustained release of FTS over a period of 1 month (Figure 1). Alternatively, FTS can be incorporated into rings via post-loading, i.e., after the 3D printing fabrication process. Results demonstrated post-loading of FTS after ring fabrication led to increase in burst release compared to rings loaded with FTS using the pre-loading process. Additionally, post-loaded rings showed faster zero-order release kinetics over 30 days in comparison to pre-loaded rings (Figure 3). Drug release kinetics can also be controlled by changing the composition and crosslink density of resin formulations. Our results showed that addition of a hydrophilic diluent (HEMA) to hydrophobic resin formulation PCL_700_-DMA lead to slower zero-order kinetics of FTS at 207 μg/day over 30 days compared to 282 μg/day obtained with FTS rings fabricated without HEMA (Figure 1). Mechanical properties of 3D printed rings can be optimized without impacting drug release kinetics of FTS. A post-fabrication UV-curing led to significant improvement in the mechanical properties of FTS loaded rings and higher FTS release rate in vitro compared to non-UV-cured rings. Collectively, these results demonstrate the flexibility of two fluticasone releasing platform technologies to achieve a controlled release kinetics and drug concentrations that can be translated to human use for treatment of EoE.

## 5. Conclusions

We have successfully demonstrated two platforms for delivery of fluticasone (FTS) for the treatment of eosinophilic esophagitis (EoE). An FTS-eluting string is an innovative approach to drug delivery that is potentially very appealing as it can be swallowed and release drug along the entire length of the esophagus. Additionally, FTS-loaded rings were successfully fabricated using 3D-DLP printing to provide controlled and prolonged drug release, though refinements will be need for more durable implantation. In vitro release studies demonstrated the ability to fine-tune drug release kinetics by changing the crosslink density of resin formulations. Post-printing UV curing improved the mechanical properties of these rings. Additionally, results showed that the process of drug loading into the rings had an effect on drug release kinetics. While there is still room to improve the drug release rate from these rings to achieve the target daily dose of 1 mg/day, and to improve the design for a more durable esophageal implantation, the approaches for esophageal drug delivery overall are very promising. To our knowledge, this is the first report of fabrication and development of string- and ring-based drug delivery devices for the treatment of EoE. Moreover, these drug delivery technologies hold the potential to also serve as a platform for treatment of other esophageal conditions, ranging from inflammatory to fibrotic to neoplastic diseases.

## Figures and Tables

**Table 1:** In vitro release kinetics of FTS from PCL strings and fabric strings (A) In vitro drug release profile of FTS loaded PCL string (2 cm L) and predicted profile for a 25 cm L string. (B) In vitro release profile of FTS loaded PCL string at varying dipping conditions. (C) In vitro release profile of FTS loaded fabric string. * Dipping Time = 2 min, Number of dips = 12, Time between dips = 30 s. ** Dipping Time = 4 min, Number of dips = 24, Time between dips = 30 s.

**(A)**					
**Dipping solution** **PLGA 50:50 Mw (kDA): Acetone (1:5)**	**Saturation solubility (FTS)** **(mg/mL)**	Amount of FTS loaded on a 2 cm string * (μg)	% **FTS released in 24 h**	**FTS released in 24 h** **(μg)**	**Predicted FTS release from 25 cm PCL string (μg)**
10.6	10.14 ± 0.15	149.01 ± 5.40	0.81 ± 0.05	1.20 ± 0.009	15
27.2	10.13 ± 1.13	250.31 ± 82.24	0.74 ± 0.15	1.85 ± 0.30	23.12
53.4	6.17 ± 1.09	4.67 ± 0.09	0.61 ± 0.01	3.50 ± 1.13	43.75
**(B)**					
**Dipping solution** **(Saturated solution of FTS in Acetone)**		Amount of FTS loaded on a 2 cm PCL string* (μg)	**% FTS released in 24 h**	**FTS released in 24 h** **(μg)**	**Predicted FTS release from 25 cm PCL string (μg)**
Dipping condition I *		935.08 ± 114.8	4.73±0.3	43.89±2.9	548.62
Dipping condition II **		1610.16±40.32	5.08±0.15	81.11±1.46	1021.93
**(C)**					
**Dipping solution** **(Saturated solution of FTS in Acetone)**		Amount of FTS loaded on a 2 cm fabric string * (μg)	**% FTS released in** **24 h**	**FTS released in 24 h** **(μg)**	**Predicted FTS release from 25 cm fabric string (μg)**
Dipping condition II **		1756.6±20.29	5.54±1.1	111.81±10.17	1397.8

**Table 2:** Composition of resin formulations (PCL_700_-DMA) and (PCL_700_-DMA/HEMA) used to 3D print rings.

Formulation	PCL_700_DMA (mole%)	HEMA (mole%)	TPO (mole%)	BLS (mole%)
PCL_700_-DMA	100	-	0.04	0.007
PCL_700_-DMA/HEMA	23	73	0.05	0.001

**Table 3:** Total amount of FTS in rings normalized to the weights of rings (n = 3), release rate of FTS at zero order kinetics (μg/day).

Formulation	Amount of FTS in Rings (mg)	FTS Burst in 24 h (%)	FTS Burst in 24 h (μg)	FTS Zero Order Release Rate (μg/day)
PCL_700_-DMA	32.11 ± 1.12	2.98 ± 0.24	958.17 ± 74.45	282.0 (R^2^ = 0.99)
PCL_700_-DMA/HEMA	31.09 ± 0.62	4.21 ± 0.14	1310.0 ± 10.18	207.0 (R^2^ = 0.98)

**Table 4:** The effect of UV-curing on 3D printed rings: Radial compression force at 10%, 20%, and 50% outer diameter compression of non-UV- and UV-cured FTS-loaded EoE rings printed with two different resin formulations (PCL_700_-DMA, PCL_700_-DMA/HEMA).

Ring Diameter Displacement (%)	Compression Force PCL_700_-DMA (non-UV) (N)	Compression Force PCL_700_-DMA (UV) (N)	Compression Force PCL_700_DMA/HEMA (non-UV) (N)	Compression force PCL_700_DMA/HEMA(UV) (N)
10	0.04 ± 0.01	0.72 ± 0.09	0.10 ± 0.03	0.93 ± 0.1
20	0.10 ± 0.01	1.22 ± 0.06	0.26 ± 0.01	1.6 ± 0.03
50	0.26 ± 0.08	1.99 ± 0.14	0.96 ± 0.02	2.9 ± 0.01

**Table 5:** Total amount of FTS in rings normalized to the weights of rings (n = 3), release rate of FTS at zero order kinetics (μg/day).

Rings	Amount of FTS in Rings (mg)	FTS Burst in 24 h (%)	FTS Burst in 24 h (μg)	FTS Zero Order Release Rate (μg/day
PCL_700_-DMA (non-UV)	32.11 ± 1.12	2.98 ± 0.24	958.17 ± 74.45	282.0 (R^2^ = 0.99)
PCL_700-_DMA (UV)	32.77 ± 1.11	3.59 ± 0.41	1179.9 ± 180.8	370.0 (R^2^ = 0.98)

**Table 6:** Total amount of FTS in rings normalized to the weights of rings (n = 3), release rate of FTS at zero order kinetics (μg/day).

Rings	FTS Loading Per Ring (mg/g)	FTS Burst in 24 h (%)	FTS burst in 24 h (μg)	FTS Zero Order Release Rate (μg/day
Pre-loaded (non-UV)	7.13	3.99 ± 0.11	280.0 ± 70.0	50.0 (R^2^ = 0.99)
Post-loaded (non-UV)	6.84	7.94 ± 0.50	550.0 ± 1.0	90.0 (R^2^ = 0.99)
Pre-loaded (UV)	11.70	3.12 ± 0.13	450.0 ± 7 0.1	237 (R^2^ = 0.978)
Post-loaded (UV)	12.56	2.78 ± 0.21	420.0 ± 10.6	270 (R^2^ = 0.98)

## Data Availability

All other data supporting the findings of this manuscript are available from the corresponding author (S.R.B.) upon reasonable request.

## Supplementary Materials

The following are available online at https://www.mdpi.com/2073-4360/13/4/557/s1.

## References

[1] Furuta G.T., Liacouras C.A., Collins M.H., Gupta S.K., Justinich C., Putnam P.E., Bonis P., Hassall E., Straumann A., Rothenberg M.E. (2007). Eosinophilic Esophagitis in Children and Adults: A Systematic Review and Consensus Recommendations for Diagnosis and Treatment: Sponsored by the American Gastroenterological Association (AGA) Institute and North American Society of Pediatric Gastroenterology, Hepatology, and Nutrition. Gastroenterology.

[2] Dellon E.S., Liacouras C.A., Molina-Infante J., Furuta G.T., Spergel J.M., Zevit N., Spechler S.J., Attwood S.E., Straumann A., Aceves S.S. (2018). Updated International Consensus Diagnostic Criteria for Eosinophilic Esophagitis: Proceedings of the AGREE Conference. Gastroenterology.

[3] Dellon E.S., Jensen E.T., Martin C.F., Shaheen N.J., Kappelman M.D. (2014). Prevalence of eosinophilic esophagitis in the United States. Clin. Gastroenterol Hepatol.

[4] Jensen E.T., Kappelman M.D., Martin C.F., Dellon E.S. (2015). Health-care utilization, costs, and the burden of disease related to eosinophilic esophagitis in the United States. Am. J. Gastroenterol..

[5] Dellon E.S., Liacouras C.A. (2014). Advances in clinical management of eosinophilic esophagitis. Gastroenterology.

[6] Cotton C.C., Eluri S., Wolf W.A., Dellon E.S. (2017). Six-Food Elimination Diet and Topical Steroids are Effective for Eosinophilic Esophagitis: A Meta-Regression. Dig. Dis. Sci..

[7] Butz B.K., Wen T., Gleich G.J., Furuta G.T., Spergel J., King E., Kramer R.E., Collins M.H., Stucke E., Mangeot C. (2014). Efficacy, dose reduction, and resistance to high-dose fluticasone in patients with eosinophilic esophagitis. Gastroenterology.

[8] Dellon E.S., Woosley J.T., Arrington A., McGee S.J., Covington J., Moist S.E., Gebhart J.H., Tylicki A.E., Shoyoye S.O., Martin C.F. (2019). Efficacy of Budesonide vs Fluticasone for Initial Treatment of Eosinophilic Esophagitis in a Randomized Controlled Trial. Gastroenterology.

[9] Dellon E.S. (2017). Management of refractory eosinophilic oesophagitis. Nat. Rev. Gastroenterol. Hepatol..

[10] Dellon E.S., Sheikh A., Speck O., Woodward K., Whitlow A.B., Hores J.M., Ivanovic M., Chau A., Woosley J.T., Madanick R.D. (2012). Viscous Topical Is More Effective Than Nebulized Steroid Therapy for Patients With Eosinophilic Esophagitis. Gastroenterology.

[11] Fillon S.A., Harris J.K., Wagner B.D., Kelly C.J., Stevens M.J., Moore W., Fang R., Schroeder S., Masterson J.C., Robertson C.E. (2012). Novel device to sample the esophageal microbiome—The esophageal string test. PLoS ONE.

[12] Furuta G.T., Kagalwalla A.F., Lee J.J., Alumkal P., Maybruck B.T., Fillon S., Masterson J.C., Ochkur S., Protheroe C., Moore W. (2013). The oesophageal string test: A novel, minimally invasive method measures mucosal inflammation in eosinophilic oesophagitis. Gut.

[13] Ackerman S.J., Kagalwalla A.F., Hirano I., Gonsalves N., Katcher P.M., Gupta S., Wechsler J.B., Grozdanovic M., Pan Z., Masterson J.C. (2019). One-Hour Esophageal String Test: A Nonendoscopic Minimally Invasive Test That Accurately Detects Disease Activity in Eosinophilic Esophagitis. Am. J. Gastroenterol..

[14] Krause J., Rosenbaum C., Grimm M., Rump A., Kessler R., Hosten N., Weitschies W. (2020). The EsoCap-system—An innovative platform to drug targeting in the esophagus. J. Control. Release.

[15] Mayer H.C., Krechetnikov R. (2012). Landau-Levich flow visualization: Revealing the flow topology responsible for the film thickening phenomena. Phys. Fluids.

[16] Gibson M., Frejlich J., Machorro R. (1985). Dip-coating method for fabricating thin photoresist films. Thin Solid Films.

[17] Brinker C.J., Frye G.C., Hurd A.J., Ashley C.S. (1991). Fundamentals of sol-gel dip coating. Thin Solid Films.

[18] Plundrich N.J., Lila M.A., Plundrich N.J., Smith A.R., Borst L.B., Snider D.B., Kaser T., Blikslager A.T., Odle J., Lila M.A. (2020). Oesophageal eosinophilia accompanies food allergy to hen egg white protein in young pigs. Clin. Exp. Allergy.

[19] Barner-Kowollik C., Bastmeyer M., Blasco E., Delaittre G., Muller P., Richter B., Wegener M. (2017). 3D Laser Micro- and Nanoprinting: Challenges for Chemistry. Angew. Chem. Int. Ed. Eng..

[20] Jungst T., Smolan W., Schacht K., Scheibel T., Groll J. (2016). Strategies and Molecular Design Criteria for 3D Printable Hydrogels. Chem. Rev..

[21] Murphy S.V., Atala A. (2014). 3D bioprinting of tissues and organs. Nat. Biotechnol..

[22] Matos F., Godina R., Jacinto C., Carvalho H., Ribeiro I., Peças P. (2019). Additive Manufacturing: Exploring the Social Changes and Impacts. Sustainability.

[23] Gao W., Zhang Y., Ramanujan D., Ramani K., Chen Y., Williams C.B., Wang C.C.L., Shin Y.C., Zhang S., Zavattieri P.D. (2015). The status, challenges, and future of additive manufacturing in engineering. Comput. Aided Des..

[24] Prasad L.K., Smyth H. (2016). 3D Printing technologies for drug delivery: A review. Drug Dev. Ind. Pharm..

[25] O’Brien C.M., Holmes B., Faucett S., Zhang L.G. (2015). Three-dimensional printing of nanomaterial scaffolds for complex tissue regeneration. Tissue Eng. Part B Rev..

[26] Piard C.M., Chen Y., Fisher J.P. (2015). Cell-Laden 3D Printed Scaffolds for Bone Tissue Engineering. Clin. Rev. Bone Miner. Metab..

[27] Dawood A., Marti Marti B., Sauret-Jackson V., Darwood A. (2015). 3D printing in dentistry. Br. Den. J..

[28] Sun J., Zhou W., Huang D., Fuh J.Y.H., Hong G.S. (2015). An Overview of 3D Printing Technologies for Food Fabrication. Food Bioprocess Technol..

[29] Ambrosi A., Pumera M. (2016). 3D-printing technologies for electrochemical applications. Chem. Soc. Rev..

[30] Melchels F.P., Feijen J., Grijpma D.W. (2010). A review on stereolithography and its applications in biomedical engineering. Biomaterials.

[31] Xing J.F., Zheng M.L., Duan X.M. (2015). Two-photon polymerization microfabrication of hydrogels: An advanced 3D printing technology for tissue engineering and drug delivery. Chem. Soc. Rev..

[32] Shirazi S.F., Gharehkhani S., Mehrali M., Yarmand H., Metselaar H.S., Adib Kadri N., Osman N.A. (2015). A review on powder-based additive manufacturing for tissue engineering: Selective laser sintering and inkjet 3D printing. Sci. Technol. Adv. Mater..

[33] Zein I., Hutmacher D.W., Tan K.C., Teoh S.H. (2002). Fused deposition modeling of novel scaffold architectures for tissue engineering applications. Biomaterials.

[34] Naito T., Nakamura M., Kaji N., Kubo T., Baba Y., Otsuka K. (2016). Three-Dimensional Fabrication for Microfluidics by Conventional Techniques and Equipment Used in Mass Production. Micromachines (Basel).

[35] Chatani S., Kloxin C.J., Bowman C.N. (2014). The power of light in polymer science: Photochemical processes to manipulate polymer formation, structure, and properties. Polym. Chem..

[36] Credi C., Fiorese A., Tironi M., Bernasconi R., Magagnin L., Levi M., Turri S. (2016). 3D Printing of Cantilever-Type Microstructures by Stereolithography of Ferromagnetic Photopolymers. ACS Appl. Mater. Interfaces.

[37] Tumbleston J.R., Shirvanyants D., Ermoshkin N., Janusziewicz R., Johnson A.R., Kelly D., Chen K., Pinschmidt R., Rolland J.P., Ermoshkin A. (2015). Continuous liquid interface production of 3D objects. Science.

[38] Bagheri A., Jin J. (2019). Photopolymerization in 3D Printing. ACS Appl. Polym. Mater..

[39] O’Neill P.F., Kent N., Brabazon D. (2017). Mitigation and control of the overcuring effect in mask projection micro-stereolithography. AIP Conf. Proc..

[40] Ge Q., Sakhaei A.H., Lee H., Dunn C.K., Fang N.X., Dunn M.L. (2016). Multimaterial 4D Printing with Tailorable Shape Memory Polymers. Sci. Rep..

[41] Sun C., Fang N., Wu D.M., Zhang X. (2005). Projection micro-stereolithography using digital micro-mirror dynamic mask. Sens. Actuators A Phys..

[42] Hwang H.H., Zhu W., Victorine G., Lawrence N., Chen S. (2018). 3d Printing: 3D-Printing of Functional Biomedical Microdevices via Light- and Extrusion-Based Approaches (Small Methods 2/2018). Small Methods.

[43] Acosta-Vélez G.F., Zhu T.Z., Linsley C.S., Wu B.M. (2018). Photocurable poly(ethylene glycol) as a bioink for the inkjet 3D pharming of hydrophobic drugs. Int. J. Pharm..

[44] Holländer J., Hakala R., Suominen J., Moritz N., Yliruusi J., Sandler N. (2018). 3D printed UV light cured polydimethylsiloxane devices for drug delivery. Int. J. Pharm..

[45] Khaled S.A., Alexander M.R., Wildman R.D., Wallace M.J., Sharpe S., Yoo J., Roberts C.J. (2018). 3D extrusion printing of high drug loading immediate release paracetamol tablets. Int. J. Pharm..

[46] Zhang J., Feng X., Patil H., Tiwari R.V., Repka M.A. (2017). Coupling 3D printing with hot-melt extrusion to produce controlled-release tablets. Int. J. Pharm..

[47] Chia H.N., Wu B.M. (2015). Recent advances in 3D printing of biomaterials. J. Biol. Eng..

[48] Baum M.M., Butkyavichene I., Churchman S.A., Lopez G., Miller C.S., Smith T.J., Moss J.A. (2015). An intravaginal ring for the sustained delivery of tenofovir disoproxil fumarate. Int. J. Pharm..

[49] Kimball A.B., Javorsky E., Ron E.S., Crowley W., Langer R. (2016). A novel approach to administration of peptides in women: Systemic absorption of a GnRH agonist via transvaginal ring delivery system. J. Control Release.

[50] Nel A., Bekker L.G., Bukusi E., Hellstrm E., Kotze P., Louw C., Martinson F., Masenga G., Montgomery E., Ndaba N. (2016). Safety, Acceptability and Adherence of Dapivirine Vaginal Ring in a Microbicide Clinical Trial Conducted in Multiple Countries in Sub-Saharan Africa. PLoS ONE.

[51] Bloomquist C.J., Mecham M.B., Paradzinsky M.D., Janusziewicz R., Warner S.B., Luft J.C., Mecham S.J., Wang A.Z., DeSimone J.M. (2018). Controlling release from 3D printed medical devices using CLIP and drug-loaded liquid resins. J. Control Release.

[52] Lyapun I.N., Andryukov B.G., Bynina M.P. (2019). HeLa Cell Culture: Immortal Heritage of Henrietta Lacks. Mol. Genet. Microbiol. Virol..

[53] Mather J., Rainville P.D., Graham K.S., Plumb R.S. (2018). A High Sensitivity UPLC/MS/MS Method for the Analysis of Fluticasone Propionate in Plasma.

[54] Quéré D. (1999). Fluid coating on a fiber. Annu. Rev. Fluid Mech..

[55] Shim E., Park J.O., Srinivasarao M. (2008). Forced coating of polypropylene fibers with non-wetting fluids: The scaling of the film thickness. Mod. Phys. Lett. B.

[56] Hollister S.J. (2006). Erratum: Porous scaffold design for tissue engineering. Nat. Mater..

[57] Kweon H., Yoo M.K., Park I.K., Kim T.H., Lee H.C., Lee H.-S., Oh J.-S., Akaike T., Cho C.-S. (2003). A novel degradable polycaprolactone networks for tissue engineering. Biomaterials.

[58] Williams J.M., Adewunmi A., Schek R.M., Flanagan C.L., Krebsbach P.H., Feinberg S.E., Hollister S.J., Das S. (2005). Bone tissue engineering using polycaprolactone scaffolds fabricated via selective laser sintering. Biomaterials.

[59] Gunatillake P.A., Adhikari R. (2003). Biodegradable synthetic polymers for tissue engineering. Eur. Cells Mater..

[60] Jansen J., Melchels F.P.W., Grijpma D.W., Feijen J. (2009). Fumaric Acid Monoethyl Ester-Functionalized Poly(d,l-lactide)/N-vinyl-2-pyrrolidone Resins for the Preparation of Tissue Engineering Scaffolds by Stereolithography. Biomacromolecules.

[61] Mu Q., Wang L., Dunn C.K., Kuang X., Duan F., Zhang Z., Qi H.J., Wang T. (2017). Digital light processing 3D printing of conductive complex structures. Addit. Manuf..

[62] Choi J.-W., Wicker R., Lee S.-H., Choi K.-H., Ha C.-S., Chung I. (2009). Fabrication of 3D biocompatible/biodegradable micro-scaffolds using dynamic mask projection microstereolithography. J. Mater. Process. Technol..

[63] Salmoria G.V., Leite J.L., Ahrens C.H., Lago A., Pires A.T.N. (2007). Rapid manufacturing of PA/HDPE blend specimens by selective laser sintering: Microstructural characterization. Polym. Test..

[64] Zhang W., Sita L.R. (2008). Highly efficient, living coordinative chain-transfer polymerization of propene with ZnEt2: Practical production of ultrahigh to very low molecular weight amorphous atactic polypropenes of extremely narrow polydispersity. J. Am. Chem. Soc..

[65] Karalekas D., Rapti D. (2002). Investigation of the processing dependence of SL solidification residual stresses. Rapid Prototyp. J..

[66] Puebla K. (2012). Effects of environmental conditions, aging, and build orientations on the mechanical properties of ASTM type I specimens manufactured via stereolithography. Rapid Prototyp. J..

[67] Stansbury J.W., Idacavage M.J. (2016). 3D printing with polymers: Challenges among expanding options and opportunities. Dent Mater..

[68] Gross B.C., Erkal J.L., Lockwood S.Y., Chen C., Spence D.M. (2014). Evaluation of 3D printing and its potential impact on biotechnology and the chemical sciences. Anal. Chem..

[69] Lee J.-Y., An J., Chua C.K. (2017). Fundamentals and applications of 3D printing for novel materials. Appl. Mater. Today.

[70] Salmoria G.V., Ahrens C.H., Fredel M., Soldi V., Pires A.T.N. (2005). Stereolithography somos 7110 resin: Mechanical behavior and fractography of parts post-cured by different methods. Polym. Test..

[71] Steyrer B., Neubauer P., Liska R., Stampfl J. (2017). Visible Light Photoinitiator for 3D-Printing of Tough Methacrylate Resins. Materials (Basel).

