# Supplementary material for: Steroid Eluting Esophageal-Targeted Drug Delivery Devices for Treatment of Eosinophilic Esophagitis
Source: Polymers (Basel). 2021 Feb 13;13(4):557. doi: 10.3390/polym13040557 (PMC7917669; doi:10.3390/polym13040557)
Supplement: Supplementary file 1 [file polymers-13-00557-s001.docx]

Supporting Information

Steroid Eluting Esophageal-Targeted Drug Delivery Devices for Treatment of Eosinophilic Esophagitis

Alka Prasher ^1^, Roopali Shrivastava ^1^, Denali Dahl ^1^, Preetika Sharma-Huynh ^2^, Panita Maturavongsadit ^1^,
Tiffany Pridgen ^3^, Allison Schorzman ^4,5^, William Zamboni ^4,5^, Jisun Ban ^4,5^, Anthony Blikslager ^3^, Evan S. Dellon ^6^ and S. Rahima Benhabbour ^1,2,^*

^1^ Joint Department of Biomedical Engineering, UNC Chapel Hill & North Carolina State University and the University of North Carolina at Chapel Hill, Chapel Hill, NC, USA; alkap@email.unc.edu (A.P.); roopalis@email.unc.edu (R.S.); ddahl@email.unc.edu (D.D.); panita@med.unc.edu (P.M.)

^2^ Division of Pharmacoengineering and Molecular Pharmaceutics, UNC Eshelman School of Pharmacy, University of North Carolina at Chapel Hill, Chapel Hill, NC, USA; preetika.sharma@unc.edu

^3^ Department of Clinical Sciences, College of Veterinary Medicine, North Carolina State University, Raleigh, NC, USA; tladams3@ncsu.edu (T.P.); anthony_blikslager@ncsu.edu (A.B.)

^4^ Division of Pharmacotherapy and Experimental Therapeutics, UNC Eshelman School of Pharmacy, University of North Carolina, Chapel Hill, NC, USA; aschorz@email.unc.edu (A.S.); zamboni@email.unc.edu (W.Z.); jsharie@email.unc.edu (J.B.)

^5^ UNC Lineberger Comprehensive Cancer Center, Carolina Institute for Nanomedicine, UNC Advanced Translational Pharmacology and Analytical Chemistry Lab; aschorz@email.unc.edu (A.S.); zamboni@email.unc.edu (W.Z.); jsharie@email.unc.edu (J.B.)

^6^ Division of Gastroenterology and Hepatology, UNC School of Medicine, University of North Carolina, Chapel Hill, NC, USA; evan_dellon@med.unc.edu

***** Correspondence: benhabs@email.unc.edu; Tel.: +1-(919)-843-6142

| **Citation:** Prasher, A.; Shrivastava, R.; Dahl, D.; Sharma-Huynh, P.; Maturavongsadit, P.; Pridgen, T.; Schorzman, A.; Zamboni, W.; Ban, J.; Blikslager, A.; et al. Steroid Eluting Esophageal-Targeted Drug Delivery Devices for Treatment of  Eosinophilic Esophagitis. *Polymers* **2021**, *13*, x. https://doi.org/10.3390/xxxxx  Academic Editor: José Miguel Ferri  Received: 25 January 2021  Accepted: 8 February 2021  Published: date  **Publisher’s Note:** MDPI stays neutral with regard to jurisdictional claims in published maps and institutional affiliations.  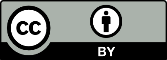  **Copyright:** © 2021 by the authors. Submitted for possible open access publication under the terms and conditions of the Creative Commons Attribution (CC BY) license (http://creativecommons.org/licenses/by/4.0/). |
| --- |

**Figure S1.** Optimization of dip coating parameters (**A**) A schematic representation of dip coating process; (**B**) A representative picture of the prototype PCL string; (**C**) A representative picture of fabric string; (**D**) The effect of retention time of strings and number of dips via repeated dipping process on the polymer mass deposited on the strings; (**E**) The effect of drying time in between the dips on mass of polymer deposited on the string. *Polymer mass deposited = M_f_ (Mass of string after dip coating) - M_i_ (Initial mass of string)*.*


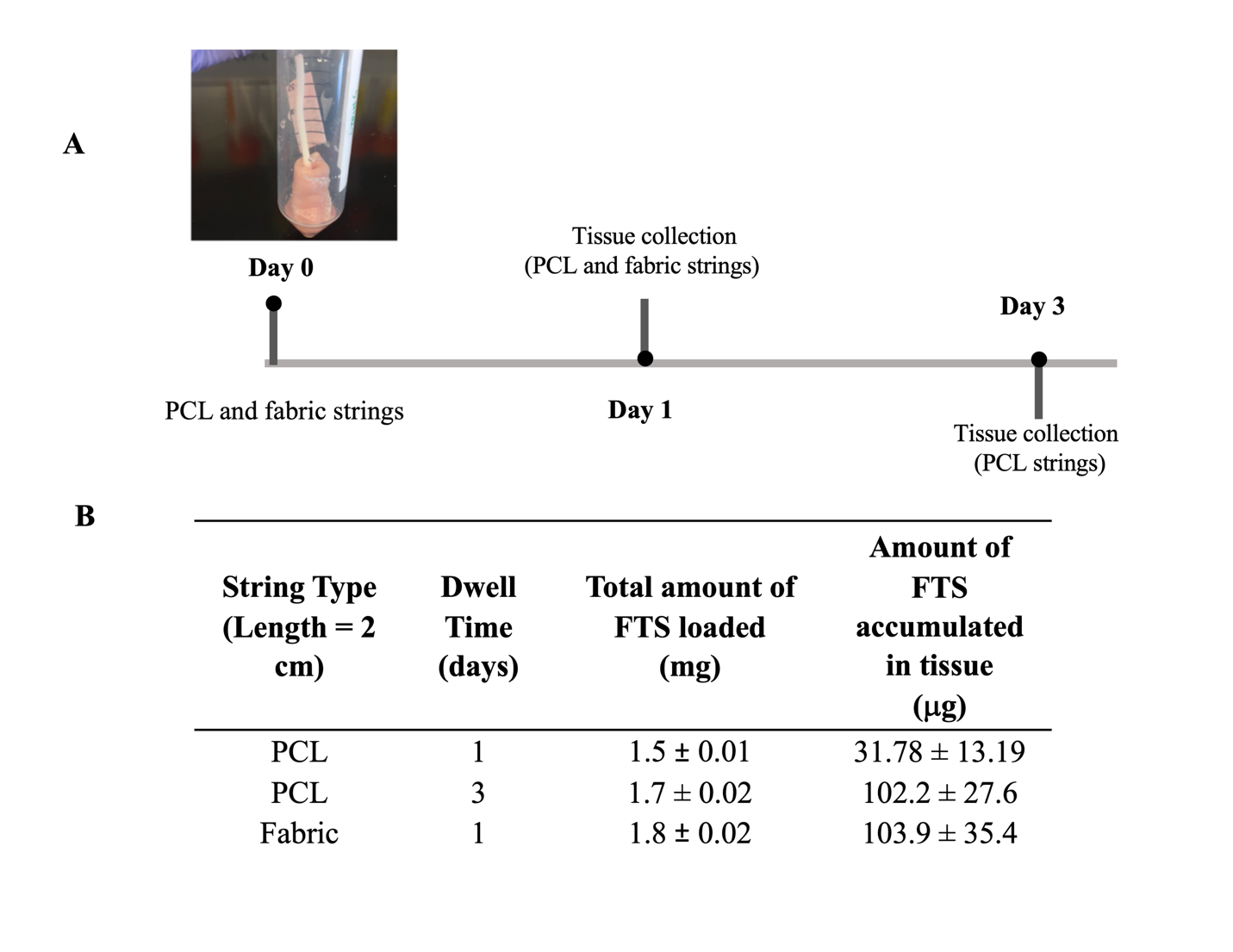


**Figure S2*.*** Ex vivo pharmacokinetic studies of FTS loaded PCL-based and fabric-based strings (**A**) Timeline of implantation of PCL and fabric strings into fresh porcine esophageal sections; (**B**) Concentration of FTS accumulated in porcine esophageal tissue at 1 and 3 days incubation quantified by LC-MS/MS analysis.

**Scheme S1*.*** Reaction scheme for synthesis of poly(caprolactone dimethacrylate) Mn = 700 gmol^−1^)*.*


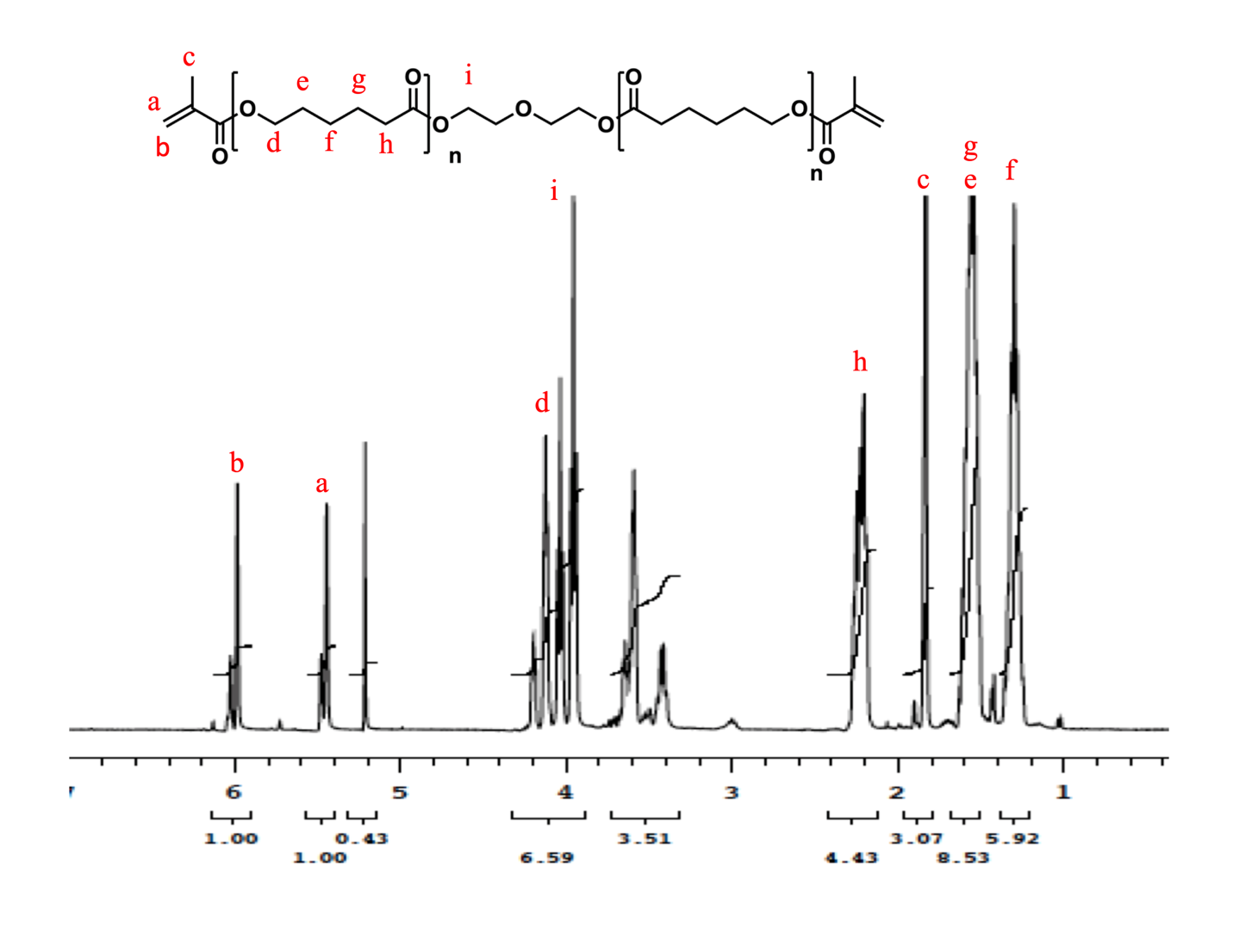


**Figure S3*.*** Representative ^1^H-NMR spectrum illustrating the successful incorporation of methacrylate functional groups in the synthesis of poly(caprolactone dimethacrylate).


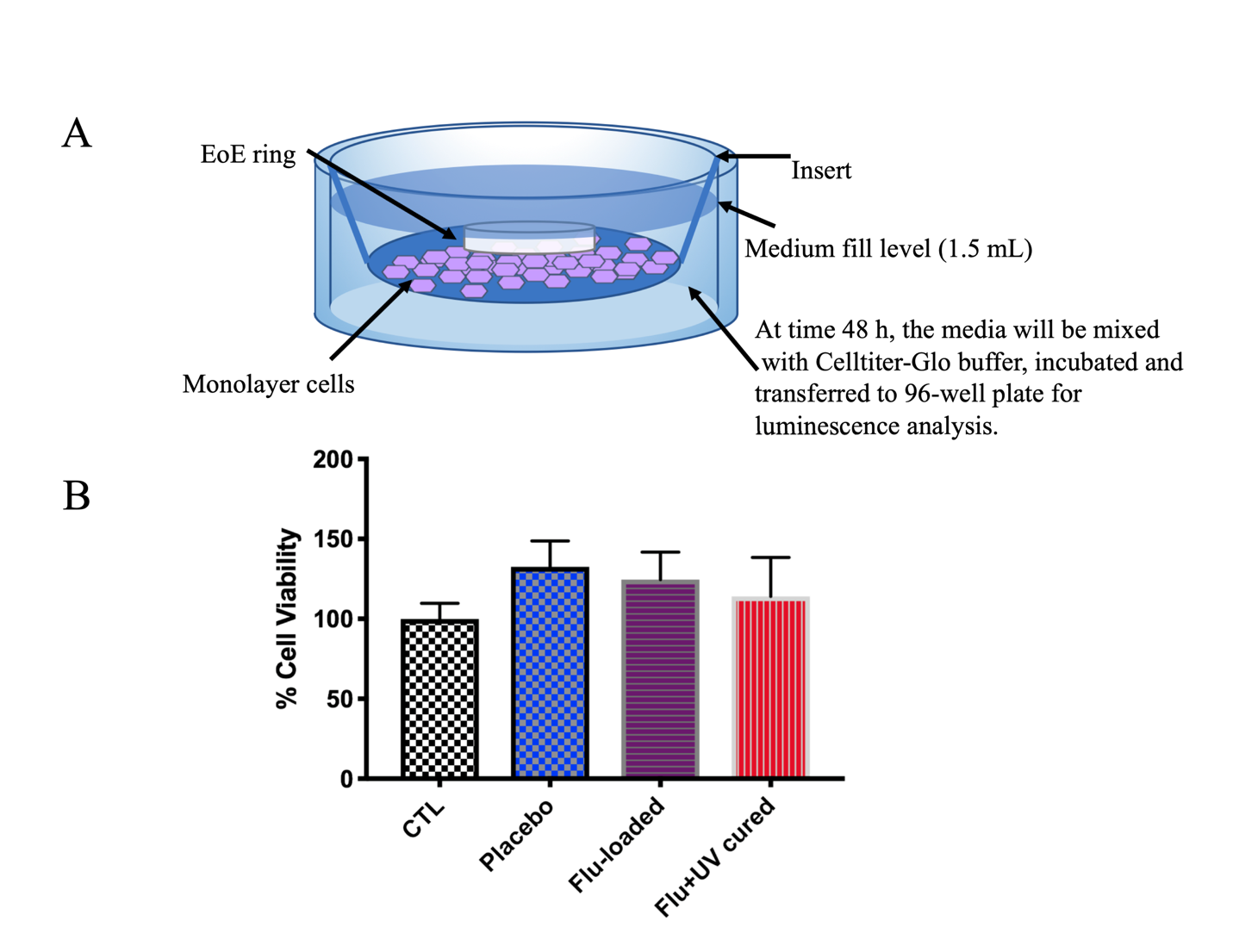


**Figure S4*.*** In vitro cytotoxicity data of 3D printed rings (**A**) A schematic representation of printed parts used in the cytotoxicity study**;** (**B**) Cytotoxicity of 3D printed disks (3 mm OD) fabricated using a PCL_700_-DMA resin formulation against HeLa cells.


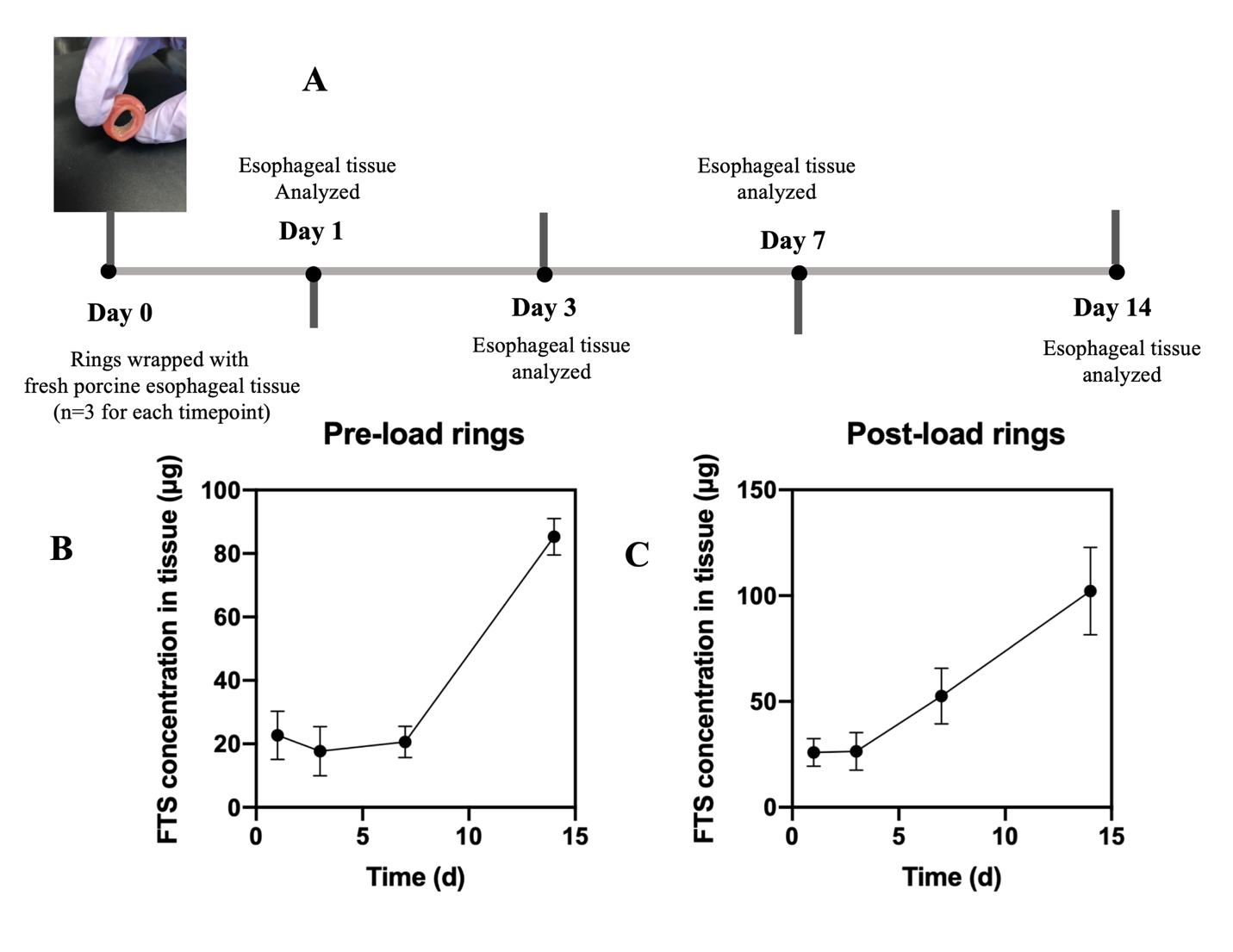


**Figure S5.** Ex vivo pharmacokinetic studies of FTS loaded rings 3D printed using a PCL_700_-DMA resin formulation via pre- and post-loading processes (**A**) Ex vivo experiments were carried out over 14 days. Esophageal tissues were wrapped around rings (n = 3) and incubated in PBS at 4 °C, and samples (1 mL) were collected at 1, 3, 7 and 14 days post-incubation; (**B**) Concentration of FTS accumulated in porcine esophageal tissue from 3D printed rings loaded via pre and post-loading at days 1, 3, 7 and 14 incubation and quantified by LC-MS/MS analysis.


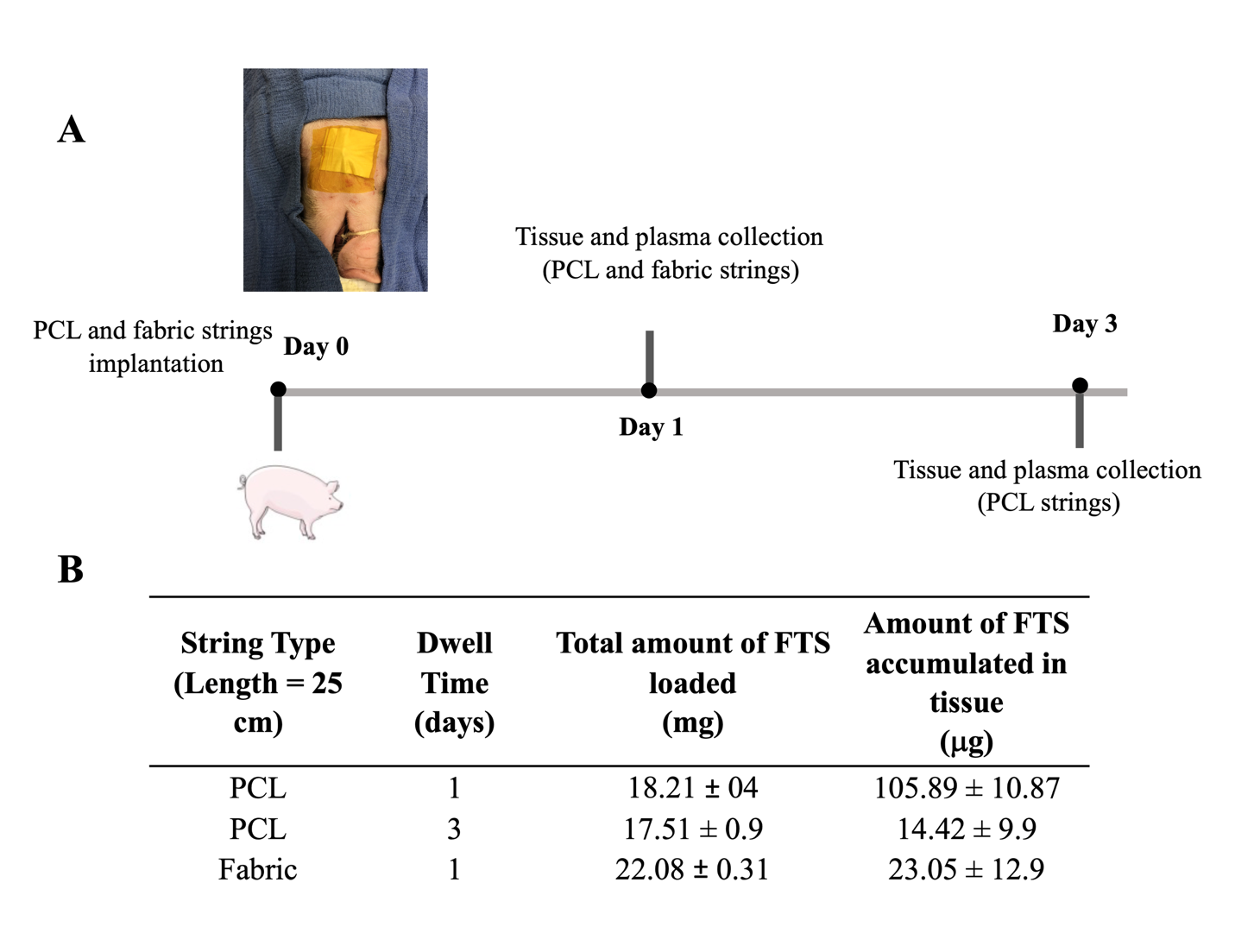


**Figure S6.** In vivo pharmacokinetic studies of FTS loaded PCL and fabric strings. (**A**) In vivo experimental design for device implantation and duration; (**B**) Concentration of FTS accumulated in porcine esophageal tissue at 1 and 3 days post-incubation and quantified by LC-MS/MS analysis.


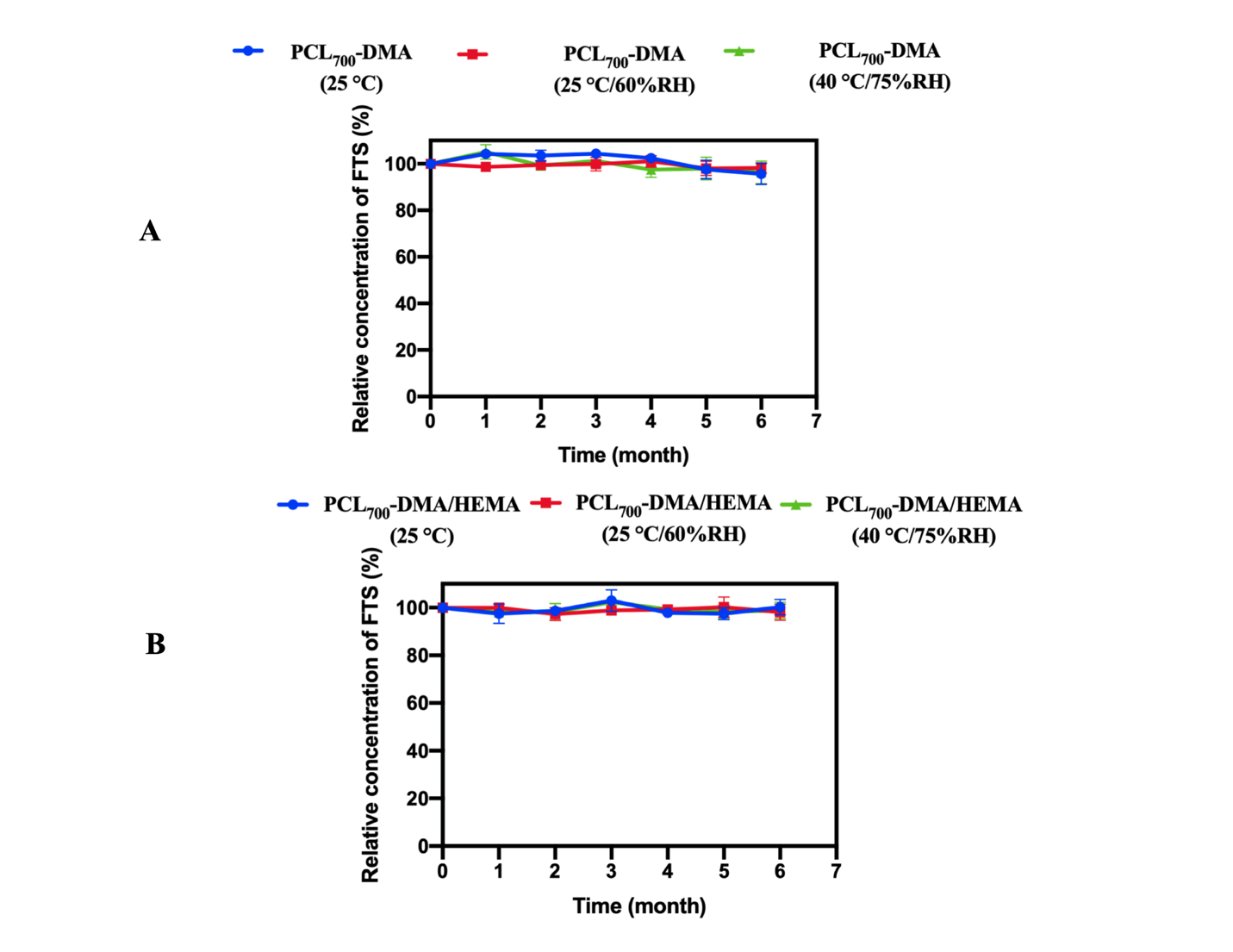


**Figure 7.** Accelerated stability studies of FTS in resin formulations (PCL_700_-DMA, PCL_700_DMA/HEMA) stored under various temperature and relative humidity conditions (RH) (25 °C, 25 °C/60% RH, 40 °C/75%RH) over 6 months (**A**) Relative percentage of FTS concentration in resin formulation PCL_700_-DMA before (day 0) and after storage at 25°C, 25 °C/60% RH, 40 °C/75%RH over 6 months; (**B**) Relative percentage of FTS concentration in resin formulation PCL_700_-DMA/HEMA before (day 0) and after storage at 25 °C, 25 °C/60% RH, 40 °C/75%RH over 6 months. FTS concentration was quantified by HPLC analysis.

**Figure S8*.*** A pictorial representation of hydrogen bonding between the hydroxyl group of HEMA and the carboxyl group in the fluticasone molecule.

**Table S1.** Effect of UV curing on Young’s modulus, Gel fraction, % swelling in chloroform and PBS of non-UV and UV cured 3D printed rings fabricated with PCL_700_-DMA and PCL_700_-DMA/HEMA resin formulations.

| **Rings** | **Young’s Modulus (Pa)** | **Gel Fraction** | **% Swelling**  **(Chloroform)** | **% Swelling**  **(PBS)** |
| --- | --- | --- | --- | --- |
| PCL_700_-DMA  (non-UV) | 8.61 ± 3.5 | 0.66 ± 0.02 | 103.4 ± 2.46 | 41.95 ± 4.95 |
| PCL_700_-DMA  (UV) | 1736.33 ± 34.8 | 0.87 ± 1.12 | 98.6 ± 1.30 | 36.43 ± 1.91 |
| PCL_700_-DMA/HEMA  (non-UV) | 10.93 ± 1.2 | 0.59 ± 2.09 | 133.21± 6.41 | 68.46 ± 4.95 |
| PCL_700_-DMA/HEMA  (UV) | 1830.9 ± 60.3 | 0.63 ± 1.21 | 102.91 ± 2.09 | 51.20 ± 1.03 |
